# The Quest for Anti-α-Synuclein Antibody Specificity—Lessons Learnt From Flow Cytometry Analysis

**DOI:** 10.3389/fneur.2022.869103

**Published:** 2022-07-15

**Authors:** Lukas Leupold, Veronika Sigutova, Elizaveta Gerasimova, Martin Regensburger, Sebastian Zundler, Friederike Zunke, Wei Xiang, Beate Winner, Iryna Prots

**Affiliations:** ^1^Department of Stem Cell Biology, University Hospital Erlangen, Friedrich-Alexander-Universität Erlangen-Nürnberg, Erlangen, Germany; ^2^Department of Molecular Neurology, University Hospital Erlangen, Friedrich-Alexander-Universität Erlangen-Nürnberg, Erlangen, Germany; ^3^Department of Medicine 1, Translational Research Center (TRC), University Hospital Erlangen, Friedrich-Alexander-Universität Erlangen-Nürnberg, Erlangen, Germany

**Keywords:** α-synuclein expression, antibody specificity, flow cytometry, antibodies, synuclein antibodies, alpha-synuclein, T cell

## Abstract

The accumulation of alpha-synuclein (aSyn) is the hallmark of a group of neurodegenerative conditions termed synucleopathies. Physiological functions of aSyn, including those outside of the CNS, remain elusive. However, a reliable and reproducible evaluation of aSyn protein expression in different cell types and especially in low-expressing cells is impeded by the existence of a huge variety of poorly characterized anti-aSyn antibodies and a lack of a routinely used sensitive detection methods. Here, we developed a robust flow cytometry-based workflow for aSyn detection and antibody validation. We test our workflow using three commercially available antibodies (MJFR1, LB509, and 2A7) in a variety of human cell types, including induced pluripotent stem cells, T lymphocytes, and fibroblasts, and provide a cell- and antibody-specific map for aSyn expression. Strikingly, we demonstrate a previously unobserved unspecificity of the LB509 antibody, while the MJFR1 clone revealed specific aSyn binding however with low sensitivity. On the other hand, we identified an aSyn-specific antibody clone 2A7 with an optimal sensitivity for detecting aSyn in a range of cell types, including those with low aSyn expression. We further utilize our workflow to demonstrate the ability of the 2A7 antibody to distinguish between physiological differences in aSyn expression in neuronal and non-neuronal cells from the cortical organoids, and in neural progenitors and midbrain dopaminergic neurons from healthy controls and in patients with Parkinson's disease who have aSyn gene locus duplication. Our results provide a proof of principle for the use of high-throughput flow cytometry-based analysis of aSyn and highlight the necessity of rigorous aSyn antibody validation to facilitate the research of aSyn physiology and pathology.

## Introduction

Pathological alpha-synuclein (aSyn) aggregation is the hallmark of a group of neurodegenerative disorders called synucleopathies. These include diseases with a high socioeconomic burden, such as Parkinson's disease (PD) and dementia with Lewy bodies (DLBs) (1). An accurate assessment of aSyn protein in various tissues is therefore crucial for the diagnosis, in-depth clinical analyses, and mechanistic investigation of synucleinopathies.

Asyn monomers are intrinsically unstructured, lacking a defined conformation, thus posing a challenge for antibody design (2, 3). Similarly, multimeric species of aSyn, such as oligomers and fibrils assume a gamut of conformations depending on formation conditions (4). Attempts to capture this diversity has led to the generation of a vast variety of antibodies currently used in synuclein research. However, many of these antibodies have not been characterized beyond the basic exploration of β-/γ-synuclein cross-reactivity using a Western blot (WB). Insufficient validation of antibody specificity has dire implications for our understanding of aSyn function as well as for diagnostic, prognostic, and therapeutic approaches in synucleopathies. Kumar et al. have recently highlighted this issue by revealing that none of the 16 tested “conformation-specific” aSyn antibodies recognized solely oligomers or fibrils, with some widely used antibodies, such as MJFR-14 even detecting monomers (5). The authors thus propose the reassessment of the previous studies using these antibodies and underline the importance of proper antibody characterization. Yet, the challenge of antibody cross-reactivity extends beyond aSyn conformations to other proteins. For instance, the commonly utilized antibody clone LB509 was raised against Lewy bodies of patients with DLB that contain a rich variety of proteins aside from aSyn, such as ubiquitin, tau, and neurofilament (6). Several recent studies have pointed out that, specifically, neurofilament light chain phosphorylated at serine 473 strongly cross-reacts with antibodies against aSyn phosphorylated at serine 129, leading to a misrepresentation of aSyn pathology (7, 8).

Moreover, even antibodies raised against recombinant aSyn can be non-specific. A widely used Syn-1 antibody, while not reactive to β- and γ-synucleins on WB, detected a 45 kDa band in aSyn null mice (9). Vojdani et al. have recently used two aSyn antibodies to probe their cross-reactivity with food products, one raised against whole aSyn and the other against a fragment mapping to residues 118–123. The whole aSyn antibody cross-reacted with nearly 50% of the tested foods; the “fragment” antibody was more restricted, but still recognized nearly 20% of the samples (10). Clone 3D5, which recognizes residues 115–122 of the aSyn protein, exhibits an extensive nuclear staining, shown to be non-specific in aSyn knockout mice (11–13). Importantly, this non-specific signal was not detected by WB, highlighting the need for cross-validation using more sensitive methods.

Flow cytometry is a viable alternative to WB for antibody detection and validation. Unlike the latter, it can detect cell-to-cell heterogeneity in protein expression lost in WB, enables multiplexing of antibodies, and is a high-throughput method. Several studies comparing the two methods have also suggested that flow cytometry is more sensitive: Kannan et al. have observed a superior specificity of flow cytometry to WB in detecting carriers for mutations in the ITGA2B gene (14). Haas et al. also reported a higher sensitivity of flow cytometry for examining the signaling pathway kinetics compared to WB (15). Furthermore, there appears to be a low correlation between protein expression determined by WB vs. flow cytometry for other commonly investigated proteins, such as Bax or ERK, although no such analysis has been performed for aSyn so far (16). To date, flow cytometry has not been routinely used for aSyn detection aside from studies utilizing fluorescently tagged transfected aSyn (17–19). Moreover, there has been little investigation into the expression of aSyn and the comparison of antibody performance across different tissues, although it is known that aSyn is expressed outside of the nervous system, is post-translationally modified in the periphery (e.g., in the blood) of patients with PD, and plays a physiological role in lymphocyte development (20–23). The characterization of aSyn expression in different cell types using validated antibodies will thus elucidate its physiology and pathology.

In this study, we establish for the first time a robust flow cytometry workflow of aSyn measurements. We test our workflow in a variety of cell types using three commercially available antibodies and provide a cell- and antibody-specific map for aSyn expression. Strikingly, we demonstrate a previously unobserved non-specificity of the LB509 antibody by successfully blocking it with other synucleins and tubulin. We determined high aSyn specificity with a low sensitivity of the MJFR1 antibody. Importantly, an anti-aSyn antibody clone 2A7 was determined to have high specificity to aSyn and to accurately detect the protein in low-expressing cell types as well as in neural progenitors and neurons with an increased aSyn gene dosage. Taken together, our results provide a proof of principle for the use of high-throughput flow cytometry-based analysis of aSyn expression and highlight the necessity of a thorough aSyn antibody validation.

## Materials and Methods

### Cells and Cell Culture

The following human induced pluripotent stem cell (hiPSC) lines from patients with PD having aSyn gene (SNCA) locus duplication (Dupl) were examined in this study: CSC-1A (Dupl 1), CSC-1D (Dupl 2, both kindly provided by Laurent Roybon, Lund University, Sweden), and SDi1-R-C11 (Dupl 3, kindly provided by Douglas Galasko, University of California, San Diego [UCSD], USA) were previously described (24–26). Dupl 1 and Dupl 2 hiPSC lines were generated from fibroblasts of the same patient with PD Dupl at Lund University. These fibroblasts originate from the Parkinson institute in Milan, Italy, where informed consent was obtained after the approval from the ethical committee. The Dupl 3 hiPSC line was reprogrammed from fibroblasts of another PD Dupl patient at the UCSD. The heterozygous SNCA duplication of the PD patient-derived lines was validated by multiplex ligation-dependent probe amplification (MLPA) analysis using the MLPA-Kits P051-C3 and P052-C2 (MRC-Holland). Control hiPSC lines were generated from fibroblasts of two age- and sex-matched healthy Caucasian individuals with no history of neurologic diseases at Universitätsklinikum Erlangen: UKERi82A-S1-017 (Ctrl 1) and UKERi33Q-R1-016 (Ctrl 2), both being a source for neural precursor cells (NPCs) as previously reported (25, 27, 28). For antibody titration and blocking experiments, hiPSC lines from two healthy Caucasian individuals were used: UKERi4L6-S1-032 or −027 and UKERi7MN-S1-010.

All experiments using hiPSC-derived cells, human fibroblasts, and human blood cells were carried out in accordance with the approval from the local Institutional Review Board (“Biobank to analyze biomarkers and generate human cellular models of neurological diseases,” No. 259_17B, Universitätsklinikum Erlangen, FAU Erlangen-Nürnberg, Erlangen, Germany) and by the Swedish work environment authority (registered under the number 20200–3211) for the work with Dupl 1 and Dupl 2 hiPSC lines. Written informed consents were received from the participants prior to inclusion into research studies.

HiPSC were cultured in mTeSR (STEMCELL Technologies) with 1% of penicillin/streptomycin (Life Technologies) at 37°C with 5% of CO_2_ on Geltrex™-coated plates (500 μg for 57 cm^2^, Thermo Fisher Scientific). To maintain the hiPSC culture, the cells were split with Accutase (Life Technologies) upon reaching a confluence of 70%. Midbrain NPC and midbrain dopaminergic neurons (mDANs) were differentiated from hiPSC by applying a small molecule-based midbrain protocol as previously described (29, 30). Cortical NPCs were differentiated from hiPSC lines using an embryoid body-based protocol as previously described (27). Cortical organoids were generated from hiPSC by applying a cerebral organoid protocol as previously described (31, 32).

Fibroblasts were obtained from skin biopsies of two healthy individuals and cultured as previously described (27, 30, 33, 34). 293T human embryonic kidney (HEK) cells (from German Collection of Microorganisms and Cell Cultures) were cultured in 10% of fetal calf serum (FCS)/Iscove's modified dulbecco's media (IMDM, Thermo Fisher Scientific) and split at 90–95% confluence. Human H4 neuroglioma cells over-expressing aSyn (H4-aSyn) under the control of a tetracycline-responsive promoter (tet-off) (kindly provided by Friederike Zunke, Universitätsklinikum Erlangen) were cultured in OptiMEM media supplemented with 1% of penicillin/streptomycin, 5% of FCS, 200 μg/ml of geniticin G418, and 200 μg/ml of hygromycin (all from Thermo Fisher Scientific) at 37°C with 5% of CO_2_ (35). For downregulating aSyn protein expression, 2 μg/ml of doxycycline was added to media for 48 h.

Venous blood was collected in heparin-containing syringes (Ratiopharm). To isolate peripheral blood mononuclear cells (PBMCs), 20 ml of blood was diluted with 20 ml of phosphate-buffered saline (PBS; Thermo Fisher Scientific) and centrifuged over a layer of 10 mL of lymphoflot (Bio-Rad) at 600 × g for 30 min at room temperature (RT). T cells were isolated from PBMC using a CD3 negative magnetic isolation (human Pan T Cell Isolation Kit, Miltenyi Biotec) according to the manufacturer's instructions and their purity was assessed by flow cytometry using anti-CD3 (a pan T-cell marker) antibody. Isolated T cells were usually more than 95% positive for CD3. T cells were cultured in Roswell Park Memorial Institute 1640 (RPMI) media (Thermo Fisher Scientific), GlutaMAX Supplement (Life Technologies) with 10% of heat-inactivated FCS (Thermo Fisher Scientific).

### Intracellular Staining and Flow Cytometry Analyses

**Figure 1:**
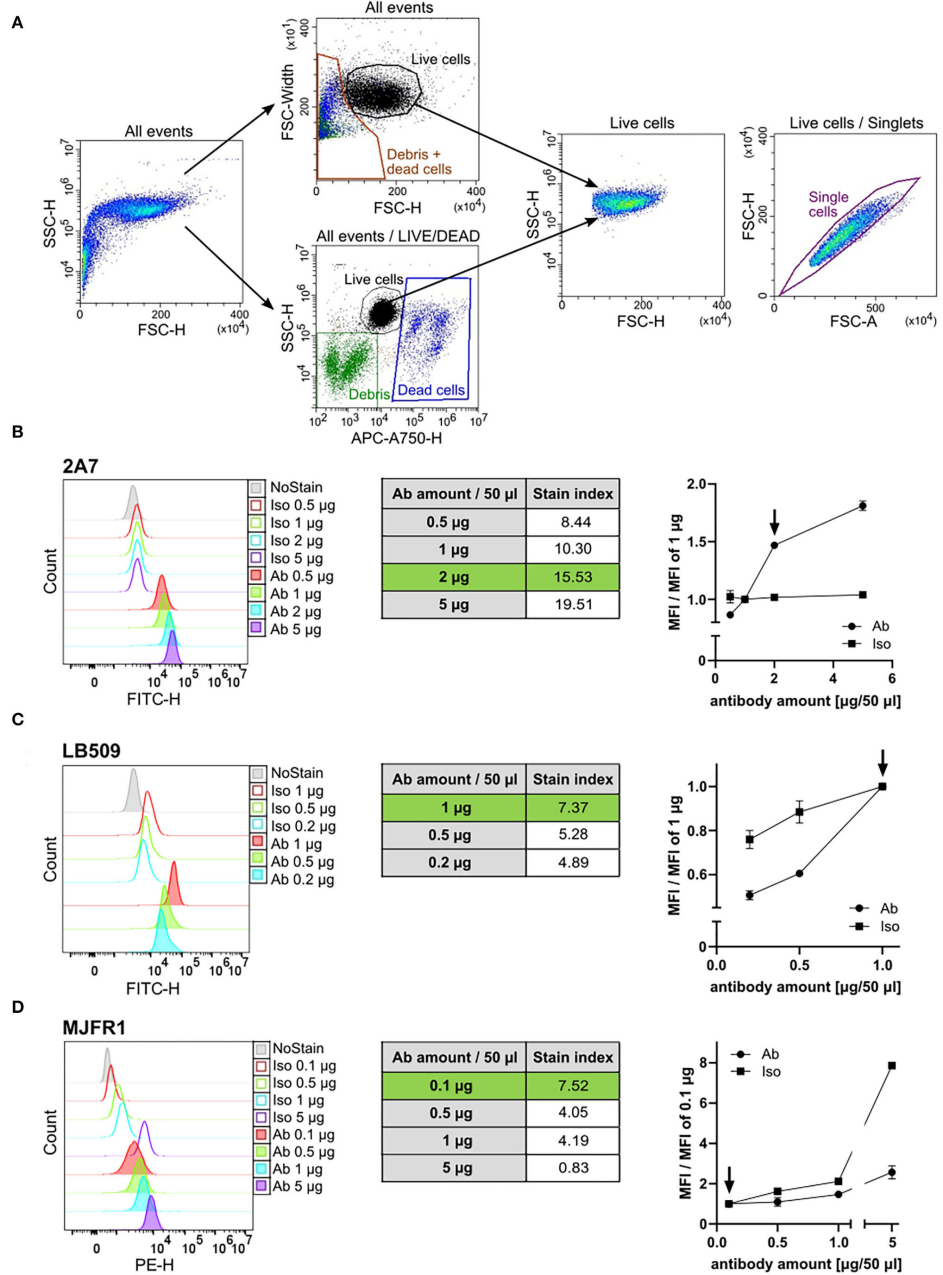
Antibody titration of the 2A7, LB509, and MJFR1. **(A)** Representative flow cytometry plots of human induced pluripotent stem cells (hiPSC) gating strategy. Two strategies for gating on living cells were applied: either an exclusion of debris and dead cells based on a forward scatter-height (FSC-H) to FSC-width plot (“All events” upper plot) or labeling of dead cells fluorescently using the Live-or-Dye™ 750/777 fixable dead cell dye (“All events/LIVE/DEAD” lower plot). Both strategies allowed for a comparable and reliable gating of living cells [“Live cells” FSC to side scatter (SSC) density plot]. Single events within living hiPSC were gated based on a pulse area to height signal ratio (“Live cells/Singlets” FSC-A to FSC-H plot). **(B–D)** Left: Histograms of the intracellular aSyn staining of hiPSC with different amounts of antibody (Ab) and the corresponding amounts of isotype control (Iso) in a total staining volume of 50 μl. Middle: Tables show the stain index (SI) of the intracellular aSyn staining with respective amounts of the 2A7 **(B)**, LB509 **(C)**, or MJFR1 **(D)** antibodies. The SI was determined for each antibody as the ratio between mean fluorescent intensity (MFI) of the positive population (antibody-stained sample) and MFI of the negative population (isotype control-stained sample) divided by two times the standard deviation (SD) of the negative population. Green rows correspond to the optimal staining-to-background conditions. Right: normalization of MFI of antibody staining signal and isotype controls to the manufacturer's recommended concentrations. Arrows indicate the optimal amount of the antibody per staining at the best signal-to-background ratio.

Two strategies for gating on living cells were applied: either an exclusion of debris and dead cells based on a forward-height (FSC-H) to FSC-width plot or labeling of dead cells fluorescently (Figure 1A). Both strategies allowed a comparable and reliable gating of living cells. To fluorescently label dead cells allowing their exclusion from flow cytometry analysis, cells were stained using the Live-or-Dye™ 750/777 Fixable Viability Staining Kit (Biotium) with the Fixable Dead Cell Dye diluted 1:1000 in PBS for 30 min at RT according to the manufacturer's instructions and washed once with PBS prior to fixation. After live/dead staining or untouched, cells were fixed with 4% of paraformaldehyde (PFA) solution in PBS for 15 min at 37°C, washed twice with PBS and either directly stained or stored at 4°C until staining. For intracellular staining, 300,000 fixed cells/staining were washed with 1 ml of FACS-PBS (PBS, 2% FCS, 0.01% NaN_3_) and permeabilized with 1x perm/wash solution (BD Biosciences) for 30 min at 4°C and incubated for 30 min at 4°C in a blocking solution (1x perm/wash with 10% of FCS and 5% of Fc receptor blocker [human TruStain FcX, BioLegend]) to avoid non-specific binding. Consequently, cells were incubated with saturating amounts of fluorescently-labeled primary antibodies in a total volume of 50 μl for 1 h at 4°C in the dark. In parallel, the respective isotype controls were applied at the corresponding amount to control for an unspecific binding. All antibodies and respective isotype controls used are listed in Table 1. After incubation with antibodies, the cells were washed twice with FACS-PBS and collected in 300 μl of FACS-PBS. Flow cytometry was performed using CytoFLEX flow cytometer (Beckman Coulter) and analyzed using FlowJo software 8.5.3 (FlowJo, LLC) or CytExpert software (Beckman Coulter). Fluorescence signals were determined using an appropriate compensation to exclude emission spectra overlap. At least 20,000 events were measured for each sample.

**Table 1:** List of antibodies used.

**Antibody**	**Clone**	**Isotype**	**Conjugate**	**Company**	**Catalog Nr**	**μg per test/dilution**	**Application**
Mouse anti-aSyn	2A7	Ms IgG1	Alexa Fluor 488 (ICC, FACS)/unconjugated (WB)	Novus Biologicals	NBP1-05194(AF488)	2/1:100/1:500	FACS/ICC/WB
Mouse anti-aSyn	LB509	Ms IgG1	Alexa Fluor 488	Santa Cruz Biotechnology	sc-58480 AF488	1/1:100	FACS/ICC
Rabbit anti-aSyn	MJFR1	Rb IgG	PE	abcam	Ab209306	0.1	FACS
Mouse anti-aSyn	syn-1	Ms IgG1	Unconjugated	BD Biosciences	610787	1:500	WB
Mouse anti-beta-III Tubulin	TU-20	Ms IgG	Alexa Fluor 405	Novus Biologicals	NB600-1018AF405	2	FACS
Rabbit anti-beta-III Tubulin	Tuj 1	Rb IgG	Unconjugated	abcam	ab18207	1:1000	ICC
Mouse IgG1 (isotype control to 2A7)	11711	Ms IgG1	Alexa Fluor 488	Novus Biologicals	IC002G	2	FACS
Mouse IgG1 (isotype control to LB509)		Ms IgG1	Alexa Fluor 488	Santa Cruz Biotechnology	sc-3890	1	FACS
Rabbit IgG (isotype control to MJFR1)	EPR25A	Rb IgG	PE	abcam	ab209478	0.1	FACS
Donkey anti-mouse IgG (H+L) (secondary)		IgG	Alexa Fluor 488	Life Technologies Corporation	A-21202	1:500	ICC
Donkey anti-rabbit IgG (H+L) (secondary)		IgG	Alexa Fluor 647	Life Technologies Corporation	A-31573	1:500	ICC
Donkey anti-mouse IgG (H+L) (secondary)		IgG	HRP	Invitrogen	SA1-100	1:10000	WB

For the titration experiments, different amounts of antibodies and their respective isotype controls were used: 0.5, 1, 2, and 5 μg per staining for the 2A7 antibody (Novus); 0.1, 0.5, 1, and 5 μg per staining for the MJFR1 antibody (ab209306, Abcam); and 0.2, 0.5, and 1 μg per staining for the LB509 antibody (Santa Cruz).

### Blocking Experiments

To assess anti-aSyn antibody specificity, blocking experiments under two different conditions varying in blocking protein amount, blocking temperature, and duration were performed systematically with all three anti-aSyn antibodies. 2A7, LB509, and MJFR1 antibodies were pre-incubated with human recombinant aSyn protein [prepared as described below based on a previously published protocol (36)] at either 300- or 600-fold higher molecular amount compared to the primary antibody at either 37°C for 1 h (for 300-fold) or RT for 2 h (for 600-fold) and subsequently applied for intracellular staining as described above.

In order to test the potential cross-reactivity of 2A7, LB509, and MJFR1 antibodies, blocking experiments with β- and γ-synucleins [prepared as described below based on a previously published protocol (36)] and pig brain-derived tubulin [prepared as previously described (37)] were performed prior to the use of the pre-incubated anti-aSyn antibodies for intracellular staining. Pre-incubation of primary antibodies with either single protein or a mixture of synucleins was performed with 600-fold molecular excess of a respective blocking protein at RT for 2 h.

Human recombinant synucleins were expressed in *Escherichia coli* and prepared as previously described (36). Briefly, *E. coli* BL21(DE3) pLysS competent cells (Novagen) were transformed with the PT7-7 construct carrying the coding region of the respective synuclein. The purification of monomeric synuclein (α, β, or γ) was performed by boiling the bacterial homogenates followed by chromatographic purification approaches including ion exchange chromatography using a Resource-Q 6 ml column (Cytiva) and size exclusion chromatography using a Superdex increase 75 10/30 column (Cytiva).

### Western Blot

H4-aSyn cells were homogenized in Tris-buffered saline (TBS, 50 mM Tris pH 7.4, 150 mM NaCl) containing 2 mM of ethylenediaminetetraacetic acid (EDTA), 1% of Triton X-100 and protease/phosphatase inhibitor cocktail (Roche) using a B. Braun Potter S Homogenizer (Sartorius AG). The homogenate was diluted at a ratio of 3:1 with 4 × RIPA buffer (200 mM of Tris/HCl pH 8.0, 600 mM of NaCl, 20 mM of EDTA, 4% of NP40, 2% of sodium deoxycholate, 0.4% SDS) and incubated on ice for 30 min to lyse the cells, followed by centrifugation at 10,000 × g for 20 min at 4°C. The supernatant was used for the rest of the procedure. Protein content was determined using a bicinchoninic acid assay (Thermo Fisher Scientific). The lysate containing 30 μg of total protein was mixed 4:1 with 5 × Laemmli sample buffer (10% of SDS, 50% of glycerol, 300 mM of Tris-HCl pH 6.8, 0.05 % of Bromophenol Blue, 100 mM of DTT), boiled for 5 min at 95°C, and separated on a 12% SDS-PAGE gel. About 7.5 ng of recombinant human aSyn was used as a positive control. The gel was blotted on a 0.2 μm of PVDF membrane using a semi-dry transfer apparatus (both Bio-Rad Laboratories).

After transfer, the blotted membranes were fixed for 30 min in 4% of PFA to increase the sensitivity of protein detection and washed thrice with TBS containing 0.1% of Tween-20 (TBST, Sigma Aldrich). To control protein loading, staining with 0.2% of Ponceau in 3% of acetic acid was performed. The membranes were next blocked with TBST containing 5% of non-fat milk and probed with primary and horseradish peroxidase-conjugated secondary antibodies in TBST containing 3% of bovine serum albumin. For visualization, chemiluminescent substrate (ECL Select, Cytiva Amersham) was applied to the membranes, and a chemiluminescent signal was detected by Gel Doc XR system (Bio-Rad Laboratories) and quantified by Fiji software (version 2.3.051; NIH). The aSyn signal was normalized to β-actin in each sample followed by the normalization to the untreated sample. Utilized antibodies and concentrations are listed in Table 1.

### Single-Cell Dissociation of Human Cortical Organoids and Staining for Flow Cytometry

HiPSC-derived cortical organoids were washed in Hank's balanced salt solution (HBSS) and dissociated into single cells using Neural Tissue Dissociation Kit (Miltenyi Biotec) as previously described (38). Cells obtained from organoids were directly filtered through a 70 μm filter. Consequently, cells were centrifuged at 500 x g for 5 min, resuspended in 1 ml of HBSS, counted, and used for surface staining with an anti-β3 tubulin (TUBB3) antibody. For surface staining, 500,000 organoid cells/staining were washed with 1 ml of FACS-PBS, blocked with 10% of FCS and 5% of Fc receptor blocker for 15 min at RT, incubated with Alexa Fluor 405-labeled anti-TUBB3 antibody (clone TU-20, Novus Biologicals) for 15 min at RT, and washed twice with FACS-PBS prior to fixation with 4% of PFA as described above and following intracellular staining.

### Bioinformatic Analysis of Protein Sequence Similarity

Local and global pairwise protein alignment was performed using the European Bioinformatics Institute tools EMBOSS Water and Needle, respectively, using the default settings (BLOSUM62 matrix, gap penalty 10, and extend penalty 0.5) (39). Annotated and reviewed protein sequences were extracted from UniProt Knowledgebase (40). The complete sequence of human aSyn (SYUA_HUMAN) was compared against pig tubulin isoforms α-1A (TBA1A_PIG), α-1B (TBA1B_PIG), and β (TBB_PIG), and against human TUBB3 (TBB3_HUMAN). The sequence alignment, residue identity, similarity, gaps, and overall scores are reported. We then compared the protein similarity between the tubulins and the epitopes of the anti-aSyn antibody clones LB509 (amino acids 115-122) and 2A7 (amino acids 61-95).

### Immunocytochemistry and Image Analysis

HiPSC-derived mDANs were fixed with 4% of PFA at RT for 10 min. Fixed cells were blocked and permeabilized for 1 h with PBS containing 10% of donkey serum (Pan Biotech), 0.3% of Triton X-100 (Sigma-Aldrich), and 5 μl of human Fc receptor blocker before adding primary antibodies (Table 1) for an incubation overnight at 4°C. Subsequently, cells were washed thrice with PBS containing 0.3% of Triton X-100 and incubated with appropriate fluorescently-labeled secondary antibodies (in case of directly-labeled primary antibodies; this procedure enhanced the signal) for 1.5 h at RT, followed by 5 min incubation with 4′, 6-diamidino-2-phenylindole (DAPI, 10 μg/ml, Sigma-Aldrich) at RT to stain the nuclei. Finally, stained cells were mounted on an object glass using Aqua-Poly/Mount (Polysciences). After drying overnight at RT in the dark, the object glasses were stored at 4°C in the dark. Images were acquired using Zeiss Observer Z1 fluorescence microscope (Zeiss). Images were quantified using the Fiji software (version 2.3.051; NIH).

### Data Analyses

In the antibody titration experiments, two following parameters were determined in order to identify the best antibody amount for an optimal staining: (1) ratio mean fluorescent intensity (MFI) = x μg MFI/MFI at the recommended amount to evaluate a fold change difference of the signal; and (2) the stain index (SI) was determined for each antibody as the ratio between MFI of the positive population (antibody-stained sample) and MFI of the negative population (isotype control-stained sample) divided by two times the standard deviation (SD) of the negative population to assess antibody staining efficiency and sensitivity. Blocking efficiency was calculated as ΔMFI between normally stained and peptide-blocked sample divided by the ΔMFI of normally stained sample to no stain control. Normalized MFI was calculated as MFI value of the stained sample divided by the MFI of the isotype control.

The quantification of aSyn immunofluorescence was performed using a custom semiautomated protocol in the Fiji software: the Tuj1 channel was thresholded using the Li method, applying a constant threshold. A selection covering the thresholded area was created and mean gray value was measured within the selection in the aSyn channel.

Data from all experiments were statistically analyzed with the GraphPad Prism version 8.3.0 (GraphPad Software). For statistical analysis, unpaired Student's *t*-test and two-way ANOVA with a *post-hoc* Sidak's test, and a significance level of *p* ≤ 0.05 were used. For all tests, Gaussian distribution was assumed. Values in the diagrams are expressed as means ± SD.

## Results

### Optimizing Antibody Amount of the 2A7 but Not LB509 or MJFR1 Improves aSyn Detection

In the present study, three different anti-aSyn antibodies (2A7, LB509, and MJFR1) were compared for their sensitivity and specificity to stain aSyn using flow cytometry as a detection method. The mouse monoclonal 2A7 antibody recognizes the aSyn region of the amino acids 61–95, known as the non-amyloid component (NAC) region (41). LB509 is a mouse monoclonal antibody recognizing an epitope located in the region encoded by amino acids 115-122 of aSyn (42). The epitope of the rabbit monoclonal MJFR1 anti-aSyn antibody was mapped to amino acids 118-123 in the C-terminus of the aSyn protein.

We first performed titration experiments to determine the amount of each anti-aSyn antibody for the best staining properties. We conducted antibody titration experiments in hiPSCs as this cell type is reported to express a low level of aSyn protein, making it suitable for determining the sensitivity of the tested antibodies (43, 44). Two strategies for gating on living cells were applied: either an exclusion of debris and dead cells based on a FSC-H to FSC-width plot or labeling of dead cells fluorescently, followed by their exclusion from the analysis (Figure 1A). Both strategies allowed a comparable and reliable gating of living cells (Figure 1A, Live cells). In the next step, single events within living hiPSCs were gated based on a pulse area to height signal ratio as shown in Figure 1A (Live cells/Singlets).

The signal intensities of the individual antibodies at amounts of 0.1–5 μg per staining in a total volume of 50 μl were compared to the amount suggested by the manufacturer; specifically, 1 μg for the antibodies 2A7 and LB509, and 0.1 μg for the antibody MJFR1. To find an optimal amount for each anti-aSyn antibody, we tested 0.5 μg and several higher doses of the antibody 2A7 (1, 2, and 5 μg), and increasing amounts for the MJFR1 antibody (0.1, 0.5, 1, and 5 μg). For the LB509 antibody, we tested several decreasing amounts (1, 0.5, and 0.2 μg). In parallel, respective isotype controls in the corresponding amounts were applied to determine whether a higher signal intensity with a larger amount of antibody will be due to better staining properties of the respective antibody or an increase in an unspecific binding. In order to identify the best antibody amount for an optimal signal intensity and the highest signal vs. background ratio, at each antibody and isotype control amount used, we determined the following: (1) SI = MFI of antibody-stained sample—MFI of isotype control-stained simple/2-times SD of the isotype control-stained sample (to assess the staining intensity and sensitivity); and (2) ratio MFI = MFI at x μg/MFI at the recommended amount (to evaluate a fold change difference of a specific antibody vs. a background staining).

At the recommended concentration, the LB509 antibody provided the signal intensity of MFI = 51,230 with SI = 7.37, whereas the 2A7 antibody revealed lower MFI of 25,778 while higher SI of 10.3 (Figures 1B,C, left and middle panels). The signal intensity of the MJFR1 antibody at 0.1 μg/staining was determined as MFI = 3,657 with SI = 7.52 (Figure 1D, left and middle panels). For the antibody 2A7, doubling the amount from 1 to 2 μg per staining enhanced the staining efficiency (SI at 2 μg = 15.53; Figure 1B, middle panel) and resulted in 1.5-fold increase in the 2 μg MFI to 1 μg MFI ratio with no changes in the isotype control binding (Figure 1B, right panel). The usage of 5 μg 2A7 antibody per staining further increased the antibody signal, whereas it did not change an unspecific binding (5 μg of MFI/1 μg of MFI equals 1.8 for the 2A7 and 1.04 for the isotype control with SI at 5 ug = 19.51; Figure 1B, middle and right panels). In order to keep an optimal relation between high signal intensity and antibody consumption, the usage of 2 μg of the 2A7 antibody per staining was set as the optimal concentration for further experiments. For LB509, reducing the antibody amount resulted in a prominent 40% and 50% drop in signal intensity for 0.5 μg and 0.2 μg of LB509 antibody per staining, respectively (ratio MFI equals 0.6 at 0.5 μg per staining and 0.5 at 0.2 μg per staining) with only moderate decrease in unspecific binding at 0.5 μg and 0.2 μg per staining with the isotype control (ratio MFI equals 0.85 at 0.5 μg and 0.75 at 0.2 μg per isotype staining; Figure 1C, right panel). In line with this, SI for the LB509 antibody at both, 0.5 μg and 0.2 μg per staining (5.28 and 4.89, respectively), was lower compared to the SI at the recommended amount (7.37, Figure 1C, middle panel). Thus, the amount recommended by the manufacturer for the LB509 antibody (1 μg per staining) was retained for further experiments. Increasing the amount of the phycoerythrin (PE)-conjugated MJFR1 antibody from 0.1 to 0.5 μg resulted in no detectable increase of the PE signal intensity but revealed a 1.6-fold increase in an unspecific binding of the respective isotype control (Figure 1D). Further increase of the MJFR1 antibody amount (1 and 5 μg) revealed much stronger increase in an unspecific binding (2.1- and 7.9-fold, respectively), while only a moderate enhancement of the MJFR1-specific signal (1.5- and 2.6-fold, respectively; Figure 1D, left and right panels). An inefficiency of staining with increasing amounts of the MJFR1 antibody was reflected by its decreasing SI at higher concentrations being 4.05, 4.19, and 0.83 at 0.5 μg, 1 μg, and 5 μg per staining, respectively (Figure 1D, middle panel). Hence, the recommended amount of 0.1 μg for the MJFR1 antibody was kept as an optimal staining amount.

### MJFR1 Exhibits Low Sensitivity for aSyn Detection

**Figure 2:**
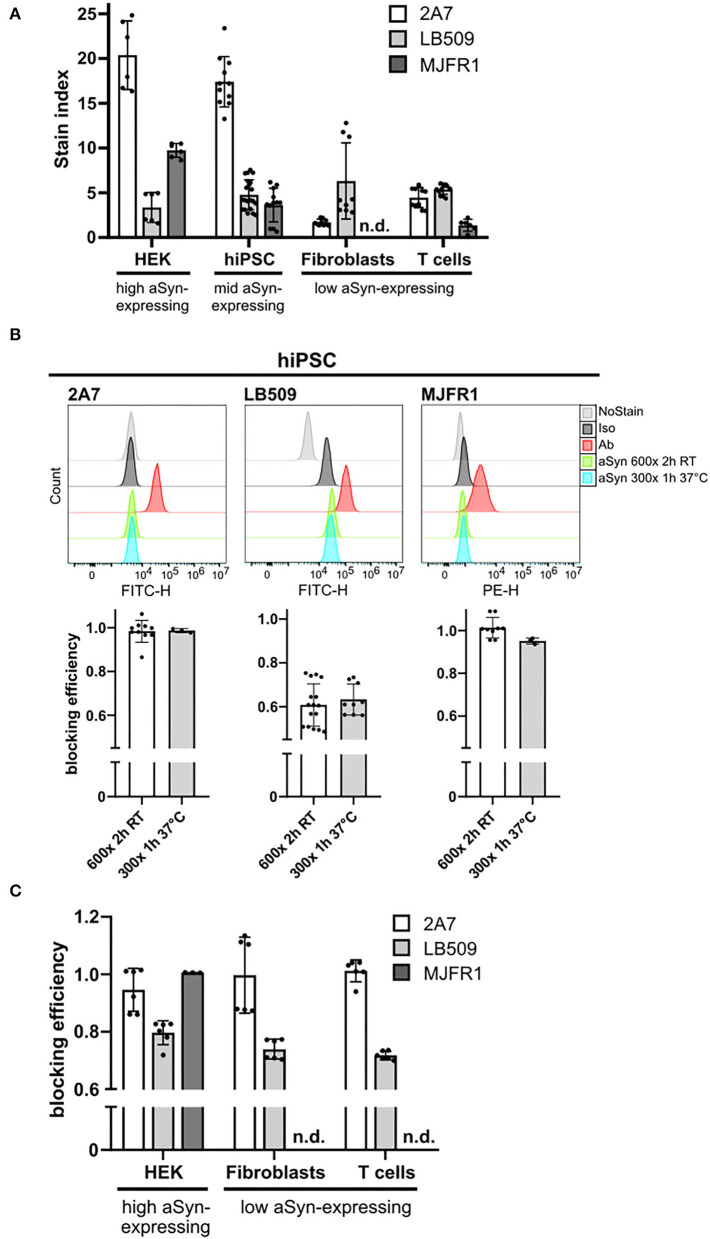
MJFR1 and 2A7 antibodies exhibit high specificity to aSyn in blocking experiments but differential sensitivity. **(A)** Stain index (SI) was calculated for three anti-aSyn antibodies used at their respective optimal amounts to quantitatively evaluate physiological aSyn protein levels in a variety of human cell types (HEK cells, hiPSC, fibroblasts, and T cells) and to compare antibody sensitivities. Six independent staining rounds of HEK cells were performed. Three hiPSC lines were used in 3–4 independent staining rounds. Fibroblasts and T cells from 2 individuals were stained in 3 independent experiments. **(B)** Top: Histograms of the intracellular aSyn staining of hiPSC, showing the signal intensities of unstained (NoStain), isotype control-stained (Iso), antibody-stained (Ab), and pre-incubated with 600- or 300-fold excess of aSyn protein either for 2 h at room temperature (RT) (aSyn 600x 2 h RT) or for 1 h at 37°C (aSyn 300x 1 h 37°C) antibody-stained cells for the AF488-labeled 2A7 and LB509 antibodies and PE-conjugated MJFR1 antibody. Bottom: Quantification of blocking efficiencies for the 2A7, LB509, and MJFR1 antibodies under both tested conditions as in the top panel. Blocking efficiency was calculated as a signal intensity difference between cells stained directly and with pre-blocked antibodies related to singal intensity difference between direct staining with a specific antibody and no stain control. 2 hiPSC lines were tested in 3 independent experiments. **(C)** Blocking of stainings with 2A7, LB509, and MJFR1 antibodies by pre-incubating them with a 600-fold molecular excess of aSyn protein for 2 h at RT in different human cell types (HEK cells, fibroblasts, and T cells). Blocking efficiency was calculated as in **(B)**. Fibroblasts and T cells from 2 individuals were stained in 3 independent experiments; 6 independent staining rounds for HEK cells were performed. Error bars represent standard deviations. n.d., not determined; RT, room temperature; h, hours.

Following the determination of the optimal antibody amounts, we compared aSyn signal intensities across multiple cell types stained with three anti-aSyn antibodies in order to assess their sensitivity for aSyn protein detection in physiological samples. We utilized HEK cells, which have been reported to have high aSyn expression (45), hiPSC with moderate levels of aSyn protein, as well as human fibroblasts and T cells expressing rather low aSyn levels (46, 47). Having the range of cell types with differential aSyn protein expression, antibody sensitivity was assessed by calculating SI for each anti-aSyn antibody at its optimal staining concentration in each cell type. In HEK cells and hiPSC, the highest SI were determined for the 2A7 antibody (20.38 and 17.32, respectively), followed by MJFR1 (9.74 and 3.64, respectively), and LB509 antibodies (3.36 and 4.77, respectively) (Figure 2A). In fibroblasts and T cells, SI values of 2A7 (1.70 and 4.46, respectively), and LB509 (6.31 and 5.37, respectively), antibodies were comparable, whereas only marginal signals could be measured for the MJFR1 antibody (undetectable in fibroblasts and 1.36 in T cells; Figure 2A). Remarkable reduction of SI for the MJFR1 antibody to its complete loss of signal, when applied to cells with moderate to low aSyn expression, indicates low sensitivity of this antibody. Whereas, SI of the 2A7 antibody was also reduced in aSyn low-expressing cells compared to HEK cells and hiPSC, the lowest aSyn signals were reliably detected by the 2A7 antibody in contrast to the MJFR1 (Figure 2A). SI of the LB509 antibody was similar in all tested cell types. Based on the 2A7 staining results, high and moderate aSyn protein expression was demonstrated in HEK cells and hiPSC, respectively, while low aSyn expression was evident in T cells and fibroblasts (Figure 2A), corroborating published results [(45–47) and the DICE Database] and indicating the best capacity of the 2A7 antibody to allow for physiological determination of aSyn protein expression.

### The 2A7 and MJFR1 Antibodies Show Higher aSyn Specificity Compared to the LB509 Antibody

Following the sensitivity testing, we performed a systematic evaluation of antibody specificity by blocking experiments using recombinant aSyn protein. These experiments were performed in hiPSC as the titration experiments demonstrated their suitability for sensitivity testing. Blocking experiments were performed under two standard pre-incubation conditions, applying either 300- or 600-fold molecular excess of the blocking protein over the antibody, 1 h or 2 h of pre-incubation time, and 37°C or RT. For all antibodies, the tested conditions for blocking experiments are listed in Table 2. The individual antibodies were pre-incubated with an excess of the recombinant human aSyn protein and subsequently applied for the aSyn staining of cells. For highly specific antibodies, no unbound antibody molecules should remain after the pre-incubation and staining of the cells should reveal no or low signal and overlap with the signal strength of the no staining control. To compare the blocking efficiency, the difference between MFI values of cells stained directly and with pre-blocked antibodies were related to the MFI difference between direct staining with a specific antibody and the no stain control.

**Table 2:** Blocking conditions for testing the specificity of anti-aSyn antibodies.

	**Conditions**
Blocking duration	1 hour (h) (for 300-fold at 37°C)
	2 h (for 600-fold at room temperature [RT])
Pre-incubation temperature	RT (for 600-fold for 2 h)
	37°C (for 300-fold for 1 h)
Protein amount	300-fold of antibody amount (for 1 h at 37°C)
	600-fold of antibody amount (for 2 h at RT)
aSyn species	Monomer
	Aggregated

Under two different conditions, the staining by the 2A7 antibody could be blocked with a similarly high efficiency by pre-incubation with 600-fold (for 2 h at RT) or 300-fold (for 1 h at 37°C) more aSyn molecules than 2A7 molecules (87 and 98%; Figure 2B, left histogram and diagram). Compared to 2A7, high blocking efficiency of the MJFR1 antibody staining was achieved by recombinant aSyn under both tested conditions (96 and 94%; Figure 2B, right histogram and diagram). Blocking efficiencies achieved under the same pre-incubating conditions for the LB509 antibody were markedly lower and revealed only 49 and 56% (Figure 2B, middle histogram and diagram), indicating its lower specificity to aSyn compared to 2A7 and MJFR1 antibodies.

We next performed systematic studies of antibody blocking applying an aSyn protein pre-incubation step in different cell types. Since both pre-incubation conditions, tested in hiPSC, resulted in a similar blocking efficiency for all three antibodies, a pre-incubation with 600-fold higher molecular aSyn amount at RT for 2 h was performed in all cell types for all three antibodies. Compared to hiPSC, in HEK cells, fibroblasts, and T cells, almost complete blocking (86, 87, 94%, respectively), was achieved with the 2A7 antibody (Figure 2C). In all cell types under the same pre-incubating conditions, the blocking efficiency for the LB509 antibody was notably lower than that of the 2A7 antibody (70–72% depending on the cell type, Figure 2C), which is slightly more effective than the blocking efficiency observed for LB509 in hiPSC. This indicates a higher specificity of the 2A7 antibody to aSyn. In accordance with a high blocking efficiency of the MJFR1 antibody staining in hiPSC, aSyn pre-incubation was effectively preventing the binding of the MJFR1 antibody to HEK cells (100% of blocking efficiency, Figure 2C). However, due to the low signal intensity of the MJFR1 antibody staining in human fibroblasts and T cells, no detectable blocking was observed in these cells (Figure 2C), indicating that MJFR1 antibody has insufficient sensitivity to detect aSyn expression at low levels.

Since the LB509 antibody was raised against Lewy bodies rather than monomeric aSyn, we tested, whether aSyn aggregates [produced *in vitro* by the previously published protocol (25)] could achieve more efficient blocking. However, the efficiencies of the LB509 antibody blocking with aSyn monomeric protein and aSyn aggregates were comparable under both tested conditions (70–80%; Supplementary Figure 1).

### The LB509 Antibody Cross-Reacts With Other Proteins of the Synuclein Family and Tubulin

**Figure 3:**
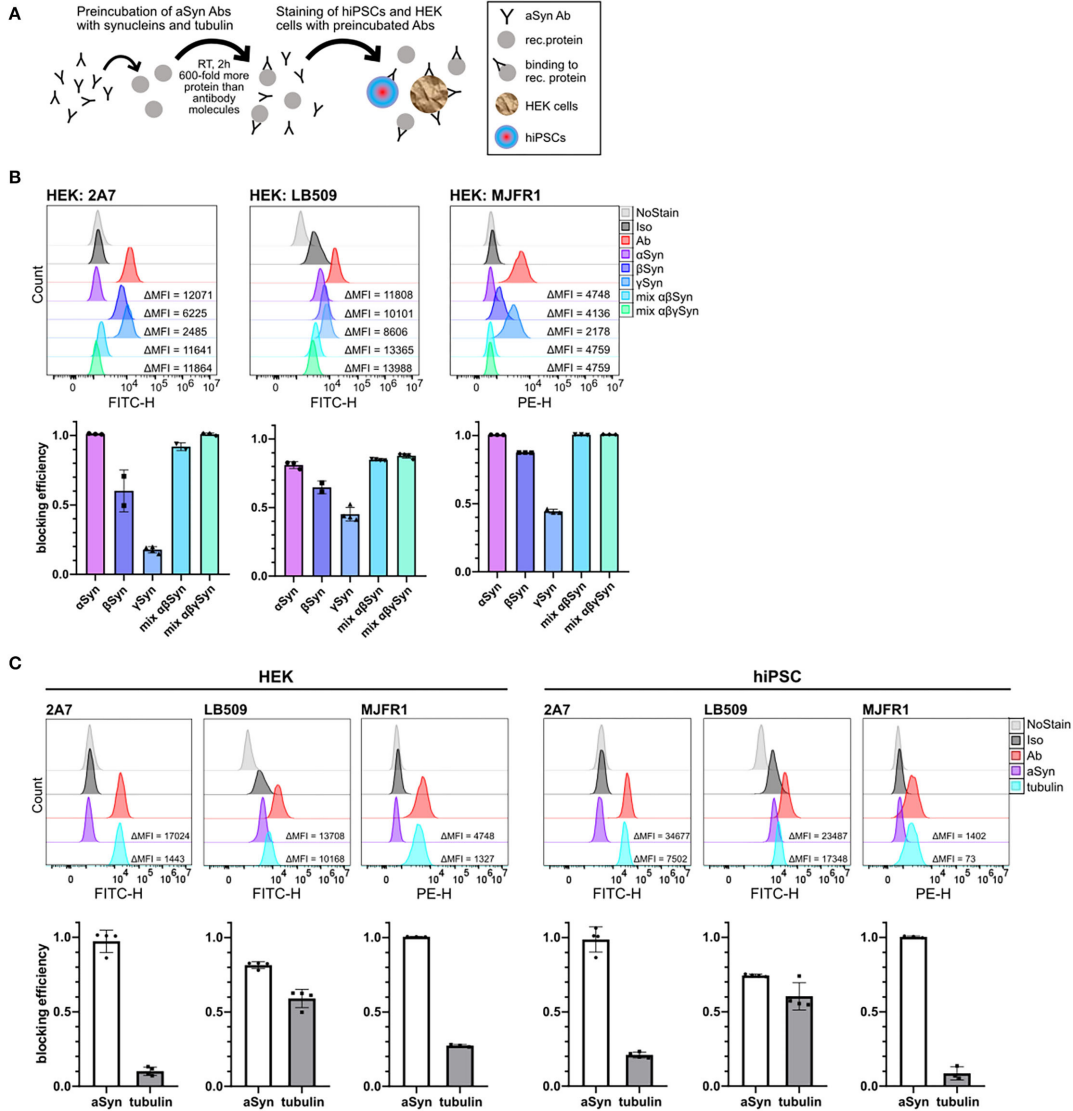
Comparison of a cross-reactivity of 2A7, LB509, and MJFR1 antibodies with different synucleins and rat tubulin. **(A)** Schematic illustration of protein blocking experiments. Anti-aSyn antibodies were pre-incubated for 2 hours at room temperature (RT) with 600-fold molecular excess of a recombinant protein and intracellular aSyn staining of hiPSC or HEK cells was performed. **(B)** In HEK cells, 2A7 and MJFR1 antibodies were efficiently blocked by pre-incubating them with aSyn, while the LB509 and MJFR1 antibodies but not the 2A7 antibody exhibit remarkable cross-reactivity with β- and γ-synucleins. Top: representative histograms of the intracellular aSyn staining of HEK cells with either AF488-conjugated 2A7 (HEK: 2A7) or LB509 antibody (HEK: LB509) or PE-conjugated MJFR1 antibody (HEK: MJFR1) before and after blocking with α-, β-, γ-synucleins (αSyn, βSyn, γSyn, respectively), or with mixtures of either α- and β-synucleins (mix αβSyn) or α-, β-, and γ-synucleins (mix α*βγ*Syn). Bottom: blocking efficiencies for each condition shown in histograms were calculated as a ratio of the difference in MFI between aSyn staining and staining with pre-blocked antibody to the difference in MFI between aSyn staining and no staining control (NoStain). Error bars represent standard deviations. **(C)** LB509, but not MJFR1 and 2A7 exhibits significant cross-reactivity with pig brain-derived tubulin in HEK cells and hiPSC. Top: histograms of the intracellular aSyn staining of HEK cells and hiPSC with 2A7, LB509, or MJFR1 antibodies before and after blocking with either aSyn (aSyn) or pig brain-derived tubulin (tubulin). Bottom: comparison of cross-reactivity of 2A7, LB509, and MJFR1 antibodies with pig brain-derived tubulin represented by blocking efficiencies. Blocking efficiency was calculated as described in **(B)**. Ab, antibody staining; Iso, staining with isotype control; MFI, mean fluorescence intensity.

To delineate the reason for the lower specificity of the LB509 antibody, we performed blocking experiments with β- and γ-synucleins, which share a sequence homology with aSyn, especially the former. The pre-incubation was done with 600-fold molecular excess of blocking protein for 2 h at RT (Figure 3A). We utilized two cell types: hiPSC, which do express moderate levels of aSyn protein, as well as HEK cells, with high aSyn expression (45), and thus both are suitable for specificity testing.

Strikingly, β-synuclein was almost as effective as aSyn in blocking the LB509 antibody staining in HEK cells (61 vs. 78%, respectively), while γ-synuclein was less potent than aSyn and blocked 41% of LB509 staining, implying low specificity of the LB509 antibody (Figure 3B, middle panel). The combination of aSyn and β-synuclein or all three synucleins blocked the LB509 antibody staining slightly more efficiently than aSyn alone (84% and 85%, respectively; Figure 3B, middle panel). In contrast, the 2A7 antibody showed only little inhibition by γ-synuclein in HEK cells (15% blocking efficiency), although a cross-reactivity with β-synuclein was observed (49% of blocking) (Figure 3B, left panel). Blocking with a mixture of aSyn and β-synuclein or all three synucleins was as effective as aSyn alone in blocking 2A7 staining (90 and 100%, respectively; Figure 3B, left panel), indicating high specificity of the 2A7 antibody toward aSyn. Similarly, to the 2A7 antibody, the MJFR1 antibody staining in HEK cells was efficiently blocked by either aSyn alone, as well as by a mixture of aSyn and β-synuclein or all three synucleins (100% blocking for each condition; Figure 3B, right panel). However, in contrast to 2A7, stronger cross-reactivity of the MJFR1 antibody was observed with β-synuclein (87% blocking efficiency) and γ-synuclein (43% blocking efficiency; Figure 3B, right panel). This data indicates the highest aSyn specificity of the 2A7 antibody followed by the MJFR1 and the lowest one of the LB509 antibody.

Additionally, we performed protein-blocking experiments with a pig brain-derived tubulin, which was intended to serve as a negative control since this protein is ubiquitously expressed. Unexpectedly, tubulin was nearly as effective as aSyn in blocking the LB509 antibody in both, hiPSC and HEK cells, further indicating only partial specificity of the LB509 antibody to aSyn (Figure 3C). In comparison, only weak blocking efficiency by tubulin could be detected for staining with the 2A7 and MJFR1 antibodies in HEK cells (7 and 26%, respectively), and hiPSC (19 and 5%, respectively; Figure 3C). Thus, the LB509 antibody is the least specific one out of three tested ones, which is in line with the results of blocking experiments with synuclein family members. Moreover, the initially observed highest signal intensity of the LB509 antibody staining could possibly be attributed to its unspecific binding to other targets besides aSyn.

To clarify why the LB509 antibody cross-reacted with tubulin, we performed a pairwise local and global sequence alignment of aSyn against pig and human tubulins using the European Bioinformatics Institute (EMBL-EBI) tools EMBOSS Water and Needle. Analysis revealed that the regions of aSyn most similar to pig tubulin β, α-1A, and α-1B chains all include the aSyn residues 115-122, which is the LB509 epitope, with all residues at least semi-conservatively preserved across the tested tubulin chains (Supplementary Figure 2). A comparable result was obtained for the human neuron-specific TUBB3, where the most similar region of aSyn spanned residues 114-131, with a 33% identity and remaining residues at least semi-conservatively preserved. Notably, the epitope of the 2A7 antibody (aSyn amino acids 61-95) exhibited lower similarity with numerous gaps/mismatches to all tested pig tubulin chains and human TUBB3 (Supplementary Figure 2), e.g., 17% and 23% identity, respectively, to α-1A, and α-1B pig tubulin chains, compared to 37.5% identity to both chains for LB509 epitope based on the Water alignment, and 0 vs. 25% identity based on Needle. Nonetheless, aSyn amino acid regions 67–82 and 74–95 did show an uninterrupted semi-conservative similarity to α-1A and α-1B pig tubulin chains, respectively, likely accounting for the low but non-zero blocking efficiency of the 2A7 antibody staining by pig tubulin.

### The 2A7 Antibody Detects Physiological Variation in aSyn Expression

**Figure 4:**
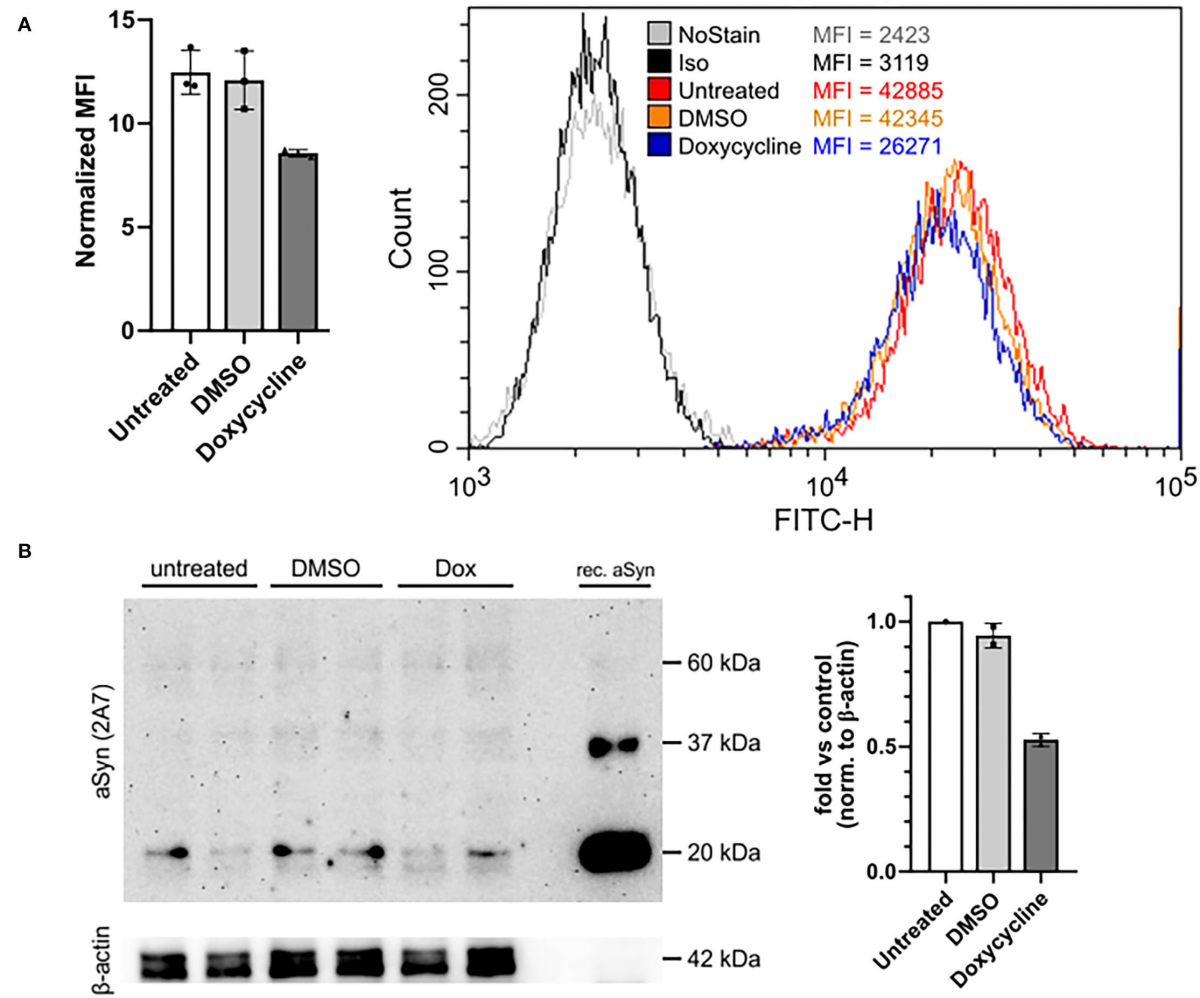
The 2A7 antibody detects physiological aSyn expression levels. **(A)** The 2A7 antibody detects biological changes of aSyn protein expression in H4 cells overexpressing aSyn under the control of a tetracycline-responsive promoter (tet-off system). Left: Comparison of normalized MFI of intracellular aSyn staining with the 2A7 antibody of H4 cells overexpressing aSyn (untreated) and treated with either diluent control (DMSO) or doxycycline (Dox). Normalized MFI was calculated as MFI value of the antibody-stained sample divided by the MFI of the isotype control staining. Right: Representative histograms of intracellular aSyn staining with the 2A7 antibody of untreated and treated with DMSO or doxycycline H4 cell overexpressing aSyn under the tet-off system. Iso, isotype control; MFI, mean fluorescence intensity. **(B)** Detection of aSyn protein levels in untreated and treated with DMSO or doxycycline (Dox) H4 cell overexpressing aSyn under the tet-off system with the 2A7 antibody by using Western blot (WB). Left: WB membrane probed with the 2A7 antibody. Two replicates per sample were loaded. Recombinant aSyn (rec. aSyn) was loaded as a positive control for aSyn protein detection. Distinct bands at the approximate size of aSyn monomer (just below 20 kDa protein marker) were detected. β-Actin was used as a loading control. Right: Quantification of the WB results revealed marked downregulation of aSyn protein level in Doxycycline-treated aSyn overexpressing H4 cells compared to DMSO-treated or untreated ones. The band intensity of aSyn monomer was normalized to β-actin in each sample followed by the normalization to the untreated sample.

Based on titration and blocking experiments, the 2A7 antibody is highly specific to aSyn and possesses the best sensitivity among three tested anti-aSyn antibodies. We next investigated if the 2A7 antibody can detect physiological changes in aSyn protein expression. For this purpose, we silenced aSyn expression in aSyn-overexpressing H4 cells with a tet-off system. In a culture media without doxycycline, dimethyl sulfoxide (DMSO, used as a diluent control) did not influence aSyn protein expression, which was determined by comparable signal intensities of the staining with the 2A7 antibody in untreated and DMSO-treated H4-aSyn cells (normalized signal-to-isotype MFI of 11.8 and 10.7, respectively; Figures 4A,B). In contrast, application of doxycycline resulted in an ~30% reduction in aSyn staining signal intensity compared to the DMSO-containing media (normalized signal-to-isotype MFI of 8.4 in doxycycline-treated H4-aSyn cells, Figure 4A), indicating the sensitivity of the 2A7 antibody to changes in aSyn expression. To confirm the flow cytometry-based detection of aSyn downregulation, we evaluated aSyn protein level in aSyn-overexpressing H4 cells before and after doxycycline treatment by WB using the 2A7 unconjugated antibody. Recombinant aSyn was loaded to serve as a positive control for aSyn detection. Distinct aSyn monomer signal (just below 20 kDa) was detected by the 2A7 antibody in all samples (Figure 4B, left panel). While no difference in aSyn monomer signal intensity between untreated and DMSO-treated aSyn-overexpressing H4 cells was obtained, a marked reduction of aSyn signal (55%) was observed in doxycycline-treated cells (Figure 4B, right panel), confirming the flow cytometry data. To reconfirm the WB aSyn detection with the 2A7 antibody, we analyzed aSyn level in the same samples by WB probed with a classically used antibody for aSyn protein detection in WB, syn-1. Similar downregulation rate of aSyn in doxycycline-treated aSyn-overexpressing H4 cells was determined by the syn-1 antibody (54%; Supplementary Figure 3, right panel). However, probing the WB membrane with syn-1 antibody revealed less prominent aSyn monomer detection and strong appearance of higher molecular weight bands in the same samples compared to the 2A7 WB detection (Supplementary Figure 3, left panel), further strengthening a high level of specificity of the 2A7 antibody.

**Figure 5:**
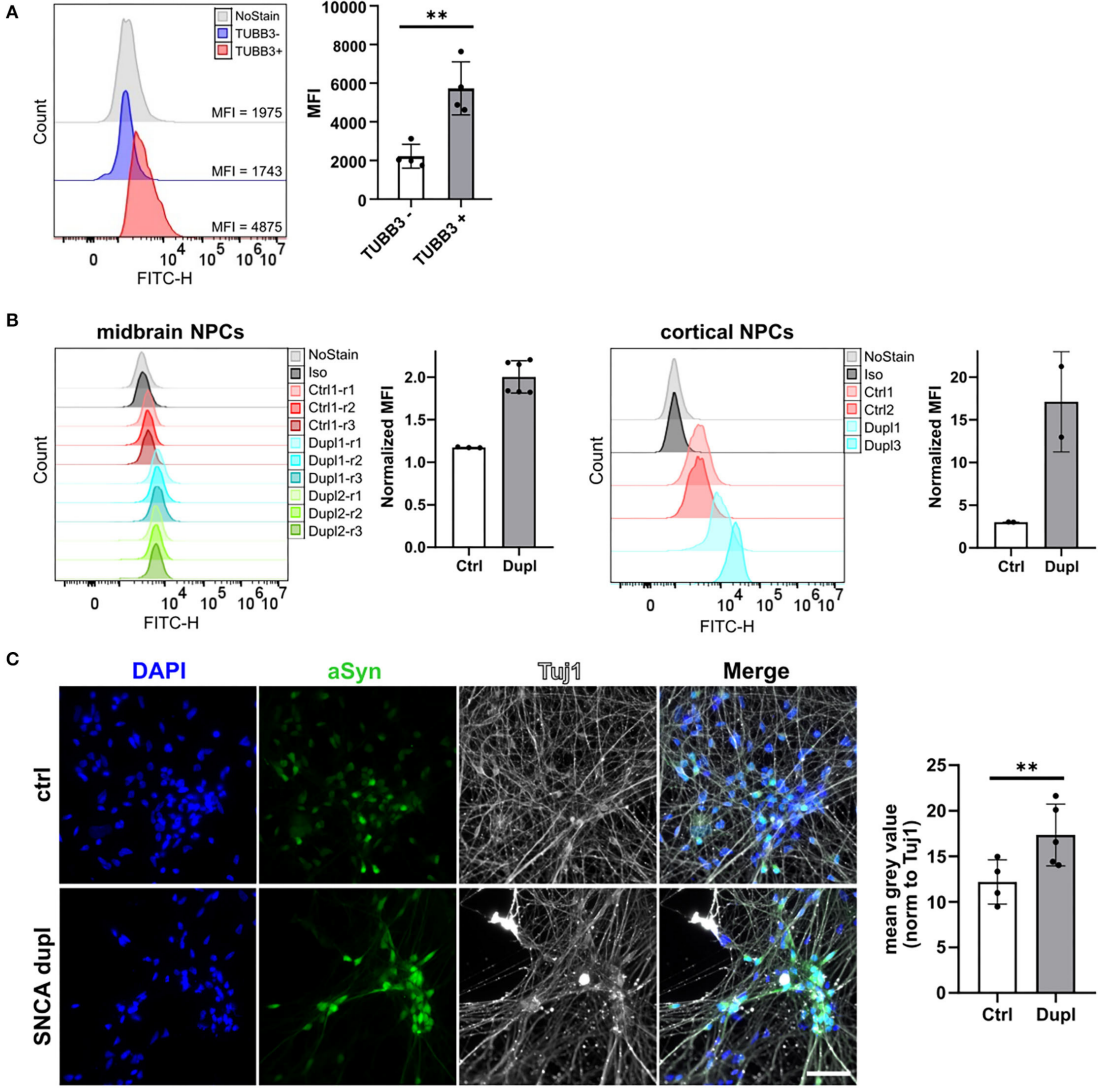
The 2A7 antibody detects aSyn expression in neural precursors and neurons. **(A)** The 2A7 antibody detects higher aSyn protein levels in neurons (TUBB3+) compared to non-neuronal cells (TUBB3-) in hiPSC-derived cortical organoids. Left: Histograms of intracellular aSyn staining of hiPSC-derived cortical organoid cells. Right: Comparison of mean fluorescence intensities (MFI) of intracellular aSyn staining in TUBB3+ and TUBB3- organoid cells. Error bars represent standard deviations. Four independent staining experiments were performed. **(B)** Intracellular aSyn staining with the 2A7 antibody in the midbrain and cortical neural precursor cells (NPCs) revealed a more robust detection of an aSyn protein increase in NPC from patients with Parkinson's disease having SNCA locus duplication (Dupl 1, 2, and 3 lines) compared to control NPC (Ctrl 1 and 2 lines). Histograms (“midbrain NPCs” and “cortical NPCs”) and quantified mean fluorescence intensities (MFI, right to each histogram) are shown. Normalized MFI values for each staining were calculated as a difference between MFI of antibody staining and of isotype control staining (Iso). **(C)** The 2A7 antibody detects a significant increase of aSyn expression in hiPSC-derived midbrain dopaminergic neurons from SNCA locus duplication in patients with Parkinson's disease (SNCA dupl) compared to control (ctrl) using immunocytochemistry. Left: Representative images of ctrl (Ctrl 1 line) and SNCA dupl hiPSC-derived (Dupl 1 line) midbrain dopaminergic neurons stained for aSyn (green) with 2A7 antibody, tubulin β3 (Tuj 1; white), and DAPI (blue). Right: Quantification of mean gray values of the 2A7 aSyn staining of ctrl and SNCA dupl midbrain dopaminergic neurons. Error bars represent standard deviations. Five independent measurements were performed. **p < 0.01, one-way ANOVA.

Finally, to prove the ability of the 2A7 antibody to detect differences in aSyn expression in physiologically relevant cell types, we applied the 2A7 antibody to control hiPSC-derived cortical organoid cells as well as to NPC and mDAN from hiPSC of healthy controls and from patients with PD Dupl. In cortical organoids, the 2A7 antibody detected a 3-fold higher aSyn expression in TUBB3-positive cells compared to TUBB3-negative cells, in agreement with the expected aSyn enrichment in neurons (Figure 5A; *p* < 0.01). Furthermore, aSyn staining with the 2A7 antibody resulted in remarkably higher signals in PD Dupl midbrain (1.7-fold) as well as cortical NPC (6.6-fold) compared to control NPC (Figure 5B), whereas only marginal, if any, increase of aSyn level in PD Dupl compared to control NPC (1.1-fold increase for midbrain and cortical) was determined by staining with the LB509 antibody (Supplementary Figure 4A). These results indicate not only the ability of the 2A7 antibody to reliably measure physiological aSyn protein levels in neuronal vs. non-neuronal cells, but furthermore, its high specificity to aSyn allow for an accurate detection of the physiological differences of aSyn protein expression in neuronal cells with SNCA dosage increase. Furthermore, the 2A7 antibody robustly differentiated aSyn protein expression between PD Dupl and control neuronal cells, indicating its high potential to reliably and specifically measure physiological aSyn expression. In immunocytochemistry, the 2A7 antibody detected a significant 1.4-fold higher aSyn signal intensity in TUBB3 (Tuj1)-positive cells from patients with PD Dupl compared to control neurons (*p* < 0.01, Figure 5C), close to the theoretical 1.5-fold increase in SNCA gene dosage. In contrast, the signal detected by the LB509 antibody in the SNCA Dupl mDAN was only 1.1-fold that of control neurons and not significantly increased with normalized staining intensities in neurons only about a half that of the 2A7 staining intensities (Supplementary Figure 4B). Overall, the results indicate the ability of the 2A7 antibody to stain aSyn in different cell types and to detect physiological differences in aSyn expression in neurons.

**Figure 6:**
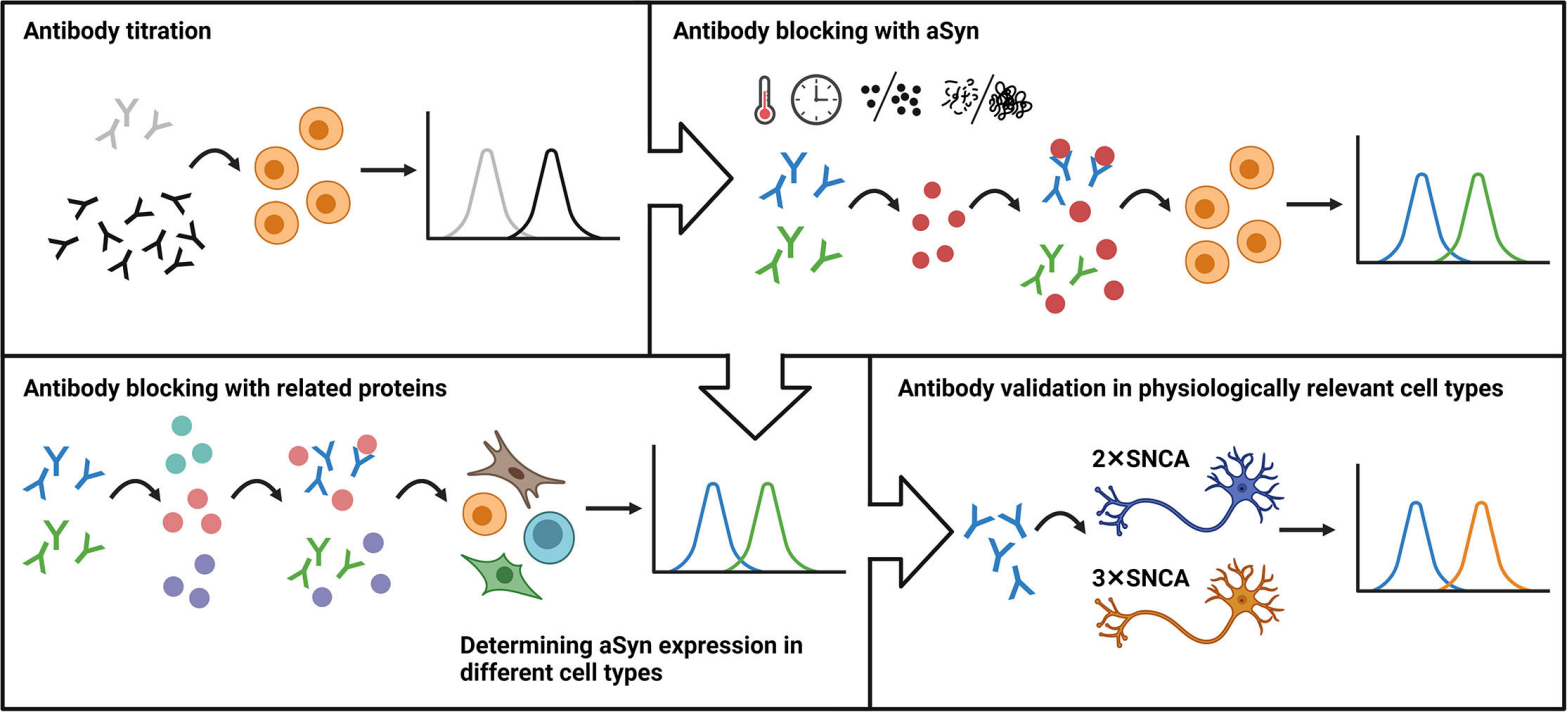
The schematic representation of the flow cytometry-based workflow for anti-aSyn antibody validation and aSyn detection. The workflow includes an antibody titration in order to optimize signal-to-noise ratio (Antibody titration), followed by blocking experiments based on the pre-incubation step of antibody with its specific target (Antibody blocking with aSyn) and related protein(s) (Antibody blocking with related proteins), evaluation antibody specificity and sensitivity in different cell types (Determining aSyn expression in different cell types), with a final confirmation of the antibody validity by testing its capability to detect physiologically-relevant protein levels (Antibody validation in physiologically-relevant cell types).

In conclusion, here, we took advantage of a classical immunological method and developed a robust flow cytometry-based workflow for aSyn detection and anti-aSyn antibody validation. The workflow includes an antibody titration in order to optimize signal-to-noise ratio, followed by blocking experiments based on the pre-incubation step of antibody with related protein(s), evaluation antibody specificity and sensitivity in different cell types, with a final confirmation of the antibody validity by testing its capability to detect physiologically relevant protein levels (Figure 6). We tested our workflow using three commercially available anti-aSyn antibodies in a variety of human cell types and provide a cell- and antibody-specific map for aSyn expression.

## Discussion

Despite the clinical importance of aSyn in neurodegenerative disorders, its pathological as well as physiological roles not only in the CNS but also in other tissues remain elusive. Understanding aSyn functions in cellular homeostasis and neurodegeneration would be facilitated by the availability of specific antibodies, compatible with widely applied techniques, such as WB, immunocytochemistry, and flow cytometry. Validation of antibodies is an integral part of translational research, particularly for biomarker discovery. Currently, a plethora of anti-aSyn antibodies is commercially available; however, most of these antibodies have not been properly characterized and validated, which is a drawback of interpreting the results on aSyn protein expression and functions. Most previous studies used WB analysis, which has limitations including lower sensitivity and the absence of routinely used isotype controls. In contrast, flow cytometry is a high-throughput method, which is more sensitive and well-controlled; however, it is barely used for aSyn expression studies. In this study, we establish a workflow for a robust characterization of aSyn antibodies by flow cytometry analysis in a variety of human cell types (Figure 6). We report the unexpected unspecificity of the widely used anti-aSyn antibody LB509, high specificity but low sensitivity for aSyn protein detection of the MJFR1 antibody, and identify the 2A7 antibody as an aSyn-specific clone capable of detecting low levels and physiological differences in aSyn expression, thus providing a valuable tool for synuclein research.

Anti-aSyn antibodies recognize a wide variety of conformational epitopes and are thus often useful only in a limited number of assays (48). Ideally, it would be necessary to screen antibodies against all possible antigens that they would encounter under experimental conditions and in different cell types. While this is currently not fully possible, the closest approximation is provided by protein microarrays, a platform by which antibody cross-reactivity against a broad range of cellular antigens can be simultaneously and quantitatively profiled (49, 50). On the other hand, flow cytometry analysis is a widely available and relatively affordable method compared to protein microarrays. Another alternative to flow cytometry is the enzyme-linked immunosorbent assay (ELISA), which has the advantage of quantitative protein content determination and is also relatively cost-effective (51). However, classical ELISA is limited in its capability to quantify only one analyte per assay. To our knowledge, there are no commercially available multiplex ELISA panels including aSyn, while flow cytometry allows relatively free multiplexing. Moreover, flow cytometry simultaneously allows for the precise identification of the population of interest by using cell type-specific markers. Additionally, ELISA is not well-suited for testing antibody performance, with its main purpose being protein content measurement. Commercial aSyn ELISA kits do not specify the antibody used (e.g., products ab260052 [Abcam], KHB0061 [Thermo Fisher Scientific], or DY1338-05 [R&D Systems]), and the preparation of homemade plates is technically challenging and labor-intensive (52). Several comparisons of ELISA with flow cytometry for cytokines also indicate a very good correlation between the two techniques, indicating that flow cytometry is sensitive to protein content despite not being strictly quantitative like ELISA (53–56). Therefore, taking into consideration the necessity to perform antibody characterization in different cell types, flow cytometry is an optimal method to validate antibodies for precise research needs.

The determination of an optimal antibody amount constitutes a key step preceding flow cytometry analysis, which is highly dependent on the density of antigen in cells (57). By implementing a titration experiment, optimal antibody amounts were determined by SI and signal-to-unspecific binding intensity ratio as 2, 1, and 0.1 μg per staining for 2A7, LB509, and MJFR1 antibodies, respectively. Notably, in case of the 2A7 antibody, an optimal concentration was different to the amount suggested by the manufacturer. Much of the discussion in recent years has centered around the frequent reports that many research antibodies fail to live up to expectations (58). Therefore, regardless of the manufacturer's instructions, the best range of dilutions needs to be determined empirically (59).

It is crucial to validate antibody specificity for particular applications, in which it will be used. Such validation exceeds the characterization performed by the manufacturer against a single or a set of purified or exogenously overexpressed targets. Indeed, in our experiments, we revealed a previously unobserved restricted specificity (shown by an incomplete blocking by recombinant aSyn and its cross-reactivity with other synucleins and tubulin) of the LB509 antibody. We observed low signal intensity (compared to other antibodies) of the MJFR1 antibody and its low sensitivity to detect marginal aSyn expression levels. While the MJFR1 antibody was efficiently blocked by recombinant aSyn, indicating its high specificity to aSyn, it cross-reacted with β- and γ-synucleins. Interestingly, a recent study showed MJFR1 cross-reactivity with 32 different food antigens, although it was explained by a high degree of peptide sequence homologies between aSyn and food products (10). A substantial cross-reactivity of the LB509 antibody with β- and γ-synucleins and tubulin was detected in our study, while the 2A7 antibody demonstrated sufficient specificity to aSyn with only slight binding to β-synuclein. Interestingly, the three tested antibodies bind to different aSyn regions: LB509 and MJFR1 antibodies recognize residues in the C-terminal domain of aSyn, while the 2A7 antibody binds to the central NAC region of the protein. This difference might influence antibody binding efficiency and specificity. A pairwise alignment of aSyn and pig tubulin/human TUBB3 protein sequences revealed that the region of aSyn most similar to TUBB3 spans residues exactly overlap with the epitopes of LB509 and MJFR1 antibodies (aSyn amino acids 115-122 and 118-123, respectively), compared to a limited similarity within the 2A7 epitope (amino acids 61-95). Whereas, no substantial binding to tubulin was observed for the MJFR1 antibody, tubulin cross-reactivity of the LB509 antibody, observed in this study, might be linked to the fact that this antibody was raised against Lewy bodies purified from the brains of patients with DLB, which contain not only aSyn, but also a number of other proteins, such as β- and γ-synucleins, tubulin, ubiquitin, tau, and neurofilament (6, 60). Notably, previous characterization of the LB509 antibody by WB has shown that this antibody failed to label the related β- and γ-synucleins (42), while in our study, a cross-reactivity of the LB509 antibody with β- and γ-synucleins was robustly demonstrated. Our data emphasize the need for more sensitive methods, such as flow cytometry, for future antibody validation.

The specificity and sensitivity of the 2A7 antibody was additionally illustrated by its ability to quantitatively determine changes of aSyn amount in the model of aSyn downregulation by doxycycline treatment in aSyn-overexpressing H4 cells using both flow cytometry and WB detection methods. Moreover, the 2A7 antibody allows for a quantitative determination of physiological aSyn protein expression in a variety of cell types: we detect high aSyn expression in HEK cells and H4 cells overexpressing aSyn, moderate to low aSyn expression in hiPSC and T cells, as well as extremely low but clearly measurable aSyn protein levels in human fibroblasts. These results can be aligned with data from previous publications and the Human Protein Atlas (45, 47, 61–63) and https://www.proteinatlas.org. In contrast, the MJFR1 antibody revealed its limited sensitivity by falling to reproducibly measure aSyn in low-expressing cell types, like fibroblasts. Taking into consideration that aSyn is an abundant CNS protein, we have demonstrated a high aSyn expression in physiologically relevant cell types by staining them with the 2A7 antibody: hiPSC-derived NPC and mDAN as well as neurons within cortical organoids. Furthermore, the 2A7 antibody was shown to be able to detect physiological differences in aSyn levels in NPC and mDAN from healthy controls and in patients with PD Dupl, making it suitable for use in synucleinopathy research. With further studies rigorously validating other aSyn antibodies, it will be possible to produce a highly verified aSyn antibody panel for a variety of assays and applications, an invaluable tool for aSyn physiology and pathology research field.

Taken together, our results establish a robust flow cytometry-based workflow for aSyn antibody testing. Specifically, we suggest to evaluating the optimal antibody dilution, before validating the specificity and sensitivity in the cell type of interest. Our data suggest that among the three tested antibodies, the 2A7 antibody is specific and sensitive enough for usage in future studies of aSyn protein expression and function in human cells. Additionally, we identify that the 2A7 antibody can detect the aSyn protein not only in cells with abundant expression, but also in low-expressing cell types, extending its utility beyond neurological research. Our findings add to the growing evidence pointing to the necessity of meticulous, manufacturer-independent antibody validation by applying sensitive and high-throughput methods, such as flow cytometry that will facilitate reliable and replicable aSyn research.

## Data Availability Statement

The original contributions presented in the study are included in the article/Supplementary Material. Further inquiries can be directed to the corresponding author.

## Ethics Statement

The studies involving human participants were reviewed and approved by the Institutional Review Board of the Friedrich-Alexander-Universität Erlangen-Nürnberg, Krankenhausstr. 12, 91054 Erlangen and by the Swedish work environment authority. The patients/participants provided their written informed consent to participate in this study.

## Author Contributions

Conceptualization: IP, VS, EG, and BW. Conduction of experiments, analysis, and interpretation of data: LL, VS, EG, SZ, FZ, WX, MR, and IP. Initial draft of manuscript: LL, VS, EG, and IP. Critical correction of manuscript and approval of final version: all authors.

## Funding

This work was funded by the Fritz Thyssen Foundation (10.19.2.024MN to IP), by the Interdisciplinary Center for Clinical Research of the University Hospital Erlangen, Germany (E30 to BW), by the German Federal Ministry of Education and Research funded TreatHSP.net consortium (01GM1905B to BW and MR and grants 01GQ113, 01GM1520A, 01EK1609B to BW), and by the Deutsche Forschungsgemeinschaft (DFG, German Research Foundation): 270949263/ GRK2162 to BW and MR and WI 3567/2-1 to BW.

## Conflict of Interest

The authors declare that the research was conducted in the absence of any commercial or financial relationships that could be construed as a potential conflict of interest.

## Publisher's Note

All claims expressed in this article are solely those of the authors and do not necessarily represent those of their affiliated organizations, or those of the publisher, the editors and the reviewers. Any product that may be evaluated in this article, or claim that may be made by its manufacturer, is not guaranteed or endorsed by the publisher.
