# Supplementary material for: The Quest for Anti-α-Synuclein Antibody Specificity—Lessons Learnt From Flow Cytometry Analysis
Source: Front Neurol. 2022 Jul 15;13:869103. doi: 10.3389/fneur.2022.869103 (PMC9334871; doi:10.3389/fneur.2022.869103)
Supplement: Supplementary file 1 [file Data_Sheet_1.PDF]

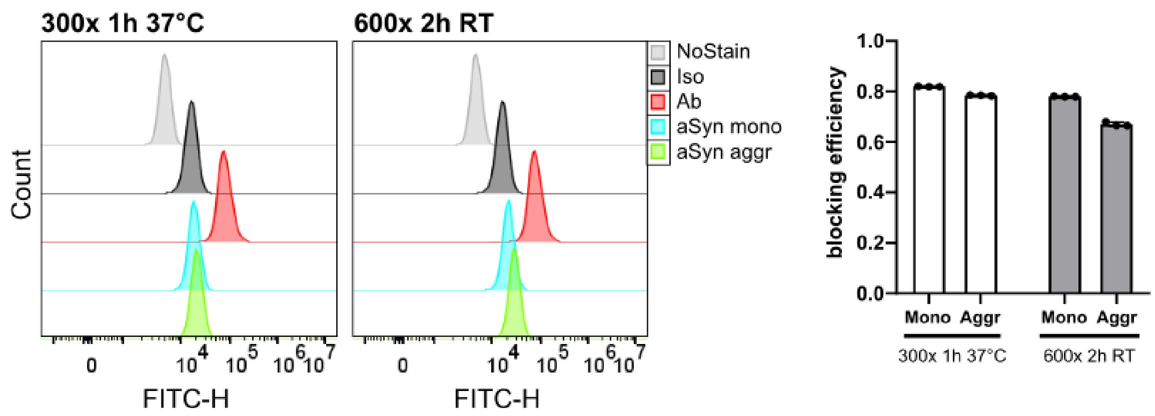

Supplementary Figure 1. Monomeric and aggregated aSyn blocks the LB509 antibody binding with similar efficiency. Blocking experiments were performed in hiPSC by pre-incubation of the LB509 antibody with either monomeric recombinant human aSyn or *in vitro* aggregated recombinant human aSyn (a mixture of oligomers and fibrils). Pre-incubation was performed with a 300- or 600-fold molecular excess of aSyn protein for either 1 hour at 37°C (300x 1h 37°C) or 2 hours at room temperature (600x 2h RT). Left: Representative histograms of the intracellular aSyn staining of hiPSC with the LB509 antibody, showing the signal intensities of unstained (NoStain), isotype control-stained (Iso), LB509 antibody-stained (Ab), and pre-incubated with monomeric (aSyn mono) or aggregated aSyn (aSyn aggr) antibody-stained cells. Right: Quantification of blocking efficiencies for the LB509 antibody staining under both tested conditions. Blocking efficiency was calculated as signal intensity difference between cells stained directly and with pre-blocked antibody related to signal intensity difference between direct staining with the antibody and no stain control. 3 independent measurements were performed.
