# Supplementary material for: The Quest for Anti-α-Synuclein Antibody Specificity—Lessons Learnt From Flow Cytometry Analysis
Source: Front Neurol. 2022 Jul 15;13:869103. doi: 10.3389/fneur.2022.869103 (PMC9334871; doi:10.3389/fneur.2022.869103)
Supplement: Supplementary file 2 [file Data_Sheet_2.docx]

**# Program: water**

#

# Aligned_sequences: 2

# 1: SYUA_HUMAN

**# 2: TBA1A_PIG**

# Matrix: EBLOSUM62

# Gap_penalty: 10.0

# Extend_penalty: 0.5

#

# Length: 108

# Identity: 24/108 (22.2%)

# Similarity: 46/108 (42.6%)

# Gaps: 16/108 (14.8%)

# Score: 40.0

#

#=======================================

SYUA_HUMAN 20 EKTKQGVAEAAGKTKEGVLYVGSKTKEGVVHGVATVAEK-TKEQVTNV-- 66

|:...|..:||.....|...:|.:..:.|:..:..:|:: |..|..:|

TBA1A_PIG 90 EQLITGKEDAANNYARGHYTIGKEIIDLVLDRIRKLADQCTGLQGFSVFH 139

SYUA_HUMAN 67 --GGAVVTGVTAVAQKTVEGAGSIAAATGFVKKDQLGKNEEGAPQEGILE 114

||...:|.|::..:.: :..:.||.:|..:...||| :.

TBA1A_PIG 140 SFGGGTGSGFTSLLMERL--------SVDYGKKSKLEFSIYPAPQ---VS 178

SYUA_HUMAN 115 DMPVDPDN 122

...|:|.|

TBA1A_PIG 179 TAVVEPYN 186

**# Program: water**

#

# Aligned_sequences: 2

# 1: SYUA_HUMAN

**# 2: TBA1B_PIG**

# Matrix: EBLOSUM62

# Gap_penalty: 10.0

# Extend_penalty: 0.5

#

# Length: 136

# Identity: 33/136 (24.3%)

# Similarity: 50/136 (36.8%)

# Gaps: 36/136 (26.5%)

# Score: 41.0

#

#=======================================

SYUA_HUMAN 37 VLYVGSKTKEGVVHGVATVAEKTKEQVT---------NVG-----GAVVT 72

:||.|....:.|...:||: |||..:. .|| ..||.

TBA1B_PIG 317 LLYRGDVVPKDVNAAIATI--KTKRSIQFVDWCPTGFKVGINYQPPTVVP 364

SYUA_HUMAN 73 G--VTAVAQKTVEGAGSIAAATGFVKKDQ---------------LGKN-E 104

| :..|.:.....:.:.|.|..:.:.|. :|:. |

TBA1B_PIG 365 GGDLAKVQRAVCMLSNTTAIAEAWARLDHKFDLMYAKRAFVHWYVGEGME 414

SYUA_HUMAN 105 EGAPQEGILEDM-PVDPDNEAYEMPSEEGYQDYEPE 139

||...|. .||| .::.|.|...:.|.||..:.|.|

TBA1B_PIG 415 EGEFSEA-REDMAALEKDYEEVGVDSVEGEGEEEGE 449

**# Program: needle**

#

# Aligned_sequences: 2

# 1: SYUA_HUMAN

**# 2: TBB_PIG**

# Matrix: EBLOSUM62

# Gap_penalty: 10.0

# Extend_penalty: 0.5

#

# Length: 540

# Identity: 13/540 ( 2.4%)

# Similarity: 20/540 ( 3.7%)

# Gaps: 495/540 (91.7%)

# Score: 15.5

#

#=======================================

SYUA_HUMAN 1 MDVFMKGLSKAKEGVVAAAEKTKQGVAEAAGKTKEGVLYVGSKTKEGVVH 50

TBB_PIG 1 -------------------------------------------------- 0

SYUA_HUMAN 51 GVATVAEKTKEQVTNVGGAVVTGVTAVAQKTVEGAGSIAAATGFVKKDQL 100

......::..|.

TBB_PIG 1 --------------------------------------MREIVHIQAGQC 12

SYUA_HUMAN 101 GKNEEGAP-QEGILEDMPVDPDNEAYEMPSEEGYQDYEPEA--------- 140

| |:.||. .|.|.::..:||.. |..|..|.:.|.

TBB_PIG 13 G-NQIGAKFWEVISDEHGIDPTG------SYHGDSDLQLERINVYYNEAA 55

SYUA_HUMAN 141 -------------------------------------------------- 140

TBB_PIG 56 GNKYVPRAILVDLEPGTMDSVRSGPFGQIFRPDNFVFGQSGAGNNWAKGH 105

SYUA_HUMAN 141 -------------------------------------------------- 140

TBB_PIG 106 YTEGAELVDSVLDVVRKESESCDCLQGFQLTHSLGGGTGSGMGTLLISKI 155

SYUA_HUMAN 141 -------------------------------------------------- 140

TBB_PIG 156 REEYPDRIMNTFSVVPSPKVSDTVVEPYNATLSVHQLVENTDETYCIDNE 205

SYUA_HUMAN 141 -------------------------------------------------- 140

TBB_PIG 206 ALYDICFRTLKLTTPTYGDLNHLVSATMSGVTTCLRFPGQLNADLRKLAV 255

SYUA_HUMAN 141 -------------------------------------------------- 140

TBB_PIG 256 NMVPFPRLHFFMPGFAPLTSRGSQQYRALTVPELTQQMFDAKNMMAACDP 305

SYUA_HUMAN 141 -------------------------------------------------- 140

TBB_PIG 306 RHGRYLTVAAVFRGRMSMKEVDEQMLNVQNKNSSYFVEWIPNNVKTAVCD 355

SYUA_HUMAN 141 -------------------------------------------------- 140

TBB_PIG 356 IPPRGLKMSATFIGNSTAIQELFKRISEQFTAMFRRKAFLHWYTGEGMDE 405

SYUA_HUMAN 141 ---------------------------------------- 140

TBB_PIG 406 MEFTEAESNMNDLVSEYQQYQDATADEQGEFEEEGEEDEA 445

**# Program: needle**

#

# Aligned_sequences: 2

# 1: SYUA_HUMAN

**# 2: TBB3_HUMAN**

# Matrix: EBLOSUM62

# Gap_penalty: 10.0

# Extend_penalty: 0.5

#

# Length: 483

# Identity: 27/483 ( 5.6%)

# Similarity: 50/483 (10.4%)

# Gaps: 376/483 (77.8%)

# Score: 17.5

#

#=======================================

SYUA_HUMAN 1 -------------------------------------------------- 0

TBB3_HUMAN 1 MREIVHIQAGQCGNQIGAKFWEVISDEHGIDPSGNYVGDSDLQLERISVY 50

SYUA_HUMAN 1 -------------------------------------------------- 0

TBB3_HUMAN 51 YNEASSHKYVPRAILVDLEPGTMDSVRSGAFGHLFRPDNFIFGQSGAGNN 100

SYUA_HUMAN 1 -------------------------------------------------- 0

TBB3_HUMAN 101 WAKGHYTEGAELVDSVLDVVRKECENCDCLQGFQLTHSLGGGTGSGMGTL 150

SYUA_HUMAN 1 -------------------------------------------------- 0

TBB3_HUMAN 151 LISKVREEYPDRIMNTFSVVPSPKVSDTVVEPYNATLSIHQLVENTDETY 200

SYUA_HUMAN 1 -------------------------------------------------- 0

TBB3_HUMAN 201 CIDNEALYDICFRTLKLATPTYGDLNHLVSATMSGVTTSLRFPGQLNADL 250

SYUA_HUMAN 1 -------------------------------------------------- 0

TBB3_HUMAN 251 RKLAVNMVPFPRLHFFMPGFAPLTARGSQQYRALTVPELTQQMFDAKNMM 300

SYUA_HUMAN 1 ------------------------------MDVFMKGLSKAKEGV----- 15

:.:..|..|...|.:

TBB3_HUMAN 301 AACDPRHGRYLTVATVFRGRMSMKEVDEQMLAIQSKNSSYFVEWIPNNVK 350

SYUA_HUMAN 16 VAAAEKTKQGVAEAAGKTKEGVLYVGSKTKEGVVHGVATVAEKTKEQVTN 65

||..:...:|: |....::|:.| .:..:.::..||.|

TBB3_HUMAN 351 VAVCDIPPRGL-------KMSSTFIGNST------AIQELFKRISEQFT- 386

SYUA_HUMAN 66 VGGAVVTGVTAVAQKTVEGAGSIAAATGFVKKDQLGKNEEGAPQEGILED 115

|:......:...|.|| .|::...|..:....::.:

TBB3_HUMAN 387 ---AMFRRKAFLHWYTGEG------------MDEMEFTEAESNMNDLVSE 421

SYUA_HUMAN 116 MPVDPDNEAYEMPSEEG--YQDYEPEA------ 140

.....|..| .||| |:|.|.|:

TBB3_HUMAN 422 YQQYQDATA----EEEGEMYEDDEEESEAQGPK 450

**# Program: water**

#

# Aligned_sequences: 2

# 1: SYUA_HUMAN

**# 2: TBB3_HUMAN**

# Matrix: EBLOSUM62

# Gap_penalty: 10.0

# Extend_penalty: 0.5

#

# Length: 18

# Identity: 6/18 (33.3%)

# Similarity: 9/18 (50.0%)

# Gaps: 0/18 ( 0.0%)

# Score: 31.0

#

#=======================================

SYUA_HUMAN 114 EDMPVDPDNEAYEMPSEE 131

:|...:.:.|.||...||

TBB3_HUMAN 426 QDATAEEEGEMYEDDEEE 443

**Supplementary File 2. α-Synuclein amino acid region 115-122 shows similarity with human and pig tubulins.** The protein sequence of human α-synuclein (SYUA_HUMAN) was pairwise aligned with pig α-1A, α-1B, and β tubulin isoforms (TBA1A_PIG, TBA1B_PIG, TBB_PIG, respectively), or with human tubulin β3 (TBB3_HUMAN). Local and global alignment was performed using the European Bioinformatics Institute (EMBL-EBI) tools EMBOSS Water and Needle, respectively, using default settings. Highlighted in cyan is the epitope of the 2A7 antibody (amino acids 61-95), in magenta the epitope of the LB509 antibody (amino acids 115-122). Higher similarity between the LB509 epitope and any tubulin compared to the 2A7 epitope with any tubulin was determined. Consensus pairwise alignment symbols: “**|**” identity; “**:**” conservative substitution (score > 1); “**.**” semi-conservative substitution (0 < score ≤ 1); “**-**” gap/mismatch.
