# Supplementary material for: The Quest for Anti-α-Synuclein Antibody Specificity—Lessons Learnt From Flow Cytometry Analysis
Source: Front Neurol. 2022 Jul 15;13:869103. doi: 10.3389/fneur.2022.869103 (PMC9334871; doi:10.3389/fneur.2022.869103)
Supplement: Supplementary file 3 [file Data_Sheet_3.pdf]

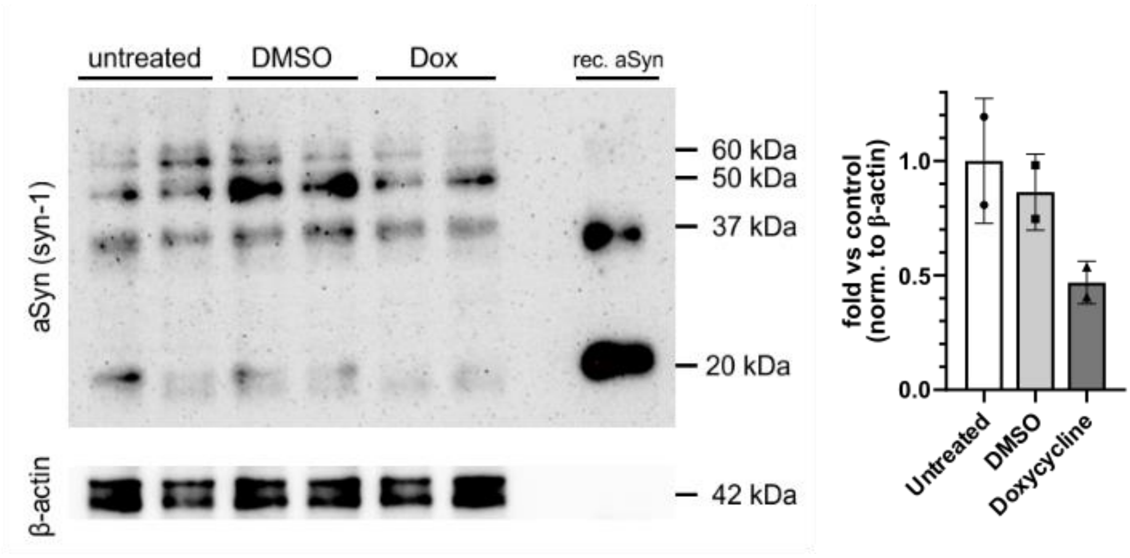

Supplementary Figure 3. Monomeric and aggregated aSyn blocks the LB509 antibody binding with similar efficiency. Detection of aSyn protein levels in untreated and treated with DMSO or doxycycline (Dox) H4 cell overexpressing aSyn under the tet-off system with the syn-1 anti-aSyn antibody by Western blot. Left: WB membrane probed with the syn-1 antibody. Two replicates per sample were loaded. Recombinant aSyn (rec. aSyn) was loaded as a positive control for aSyn protein detection. Bands just below 20 kDa protein marker correspond to aSyn monomers. β-Actin was used as a loading control. Right: Quantification of the WB results revealed marked downregulation of aSyn protein level in Doxycycline-treated aSyn overexpressing H4 cells compared to DMSO-treated or untreated ones. The aSyn monomer band intensity was normalised to β-actin in each sample followed by the normalization to the DMSO-treated sample.
