# Supplementary material for: The Quest for Anti-α-Synuclein Antibody Specificity—Lessons Learnt From Flow Cytometry Analysis
Source: Front Neurol. 2022 Jul 15;13:869103. doi: 10.3389/fneur.2022.869103 (PMC9334871; doi:10.3389/fneur.2022.869103)
Supplement: Supplementary file 4 [file Data_Sheet_4.pdf]

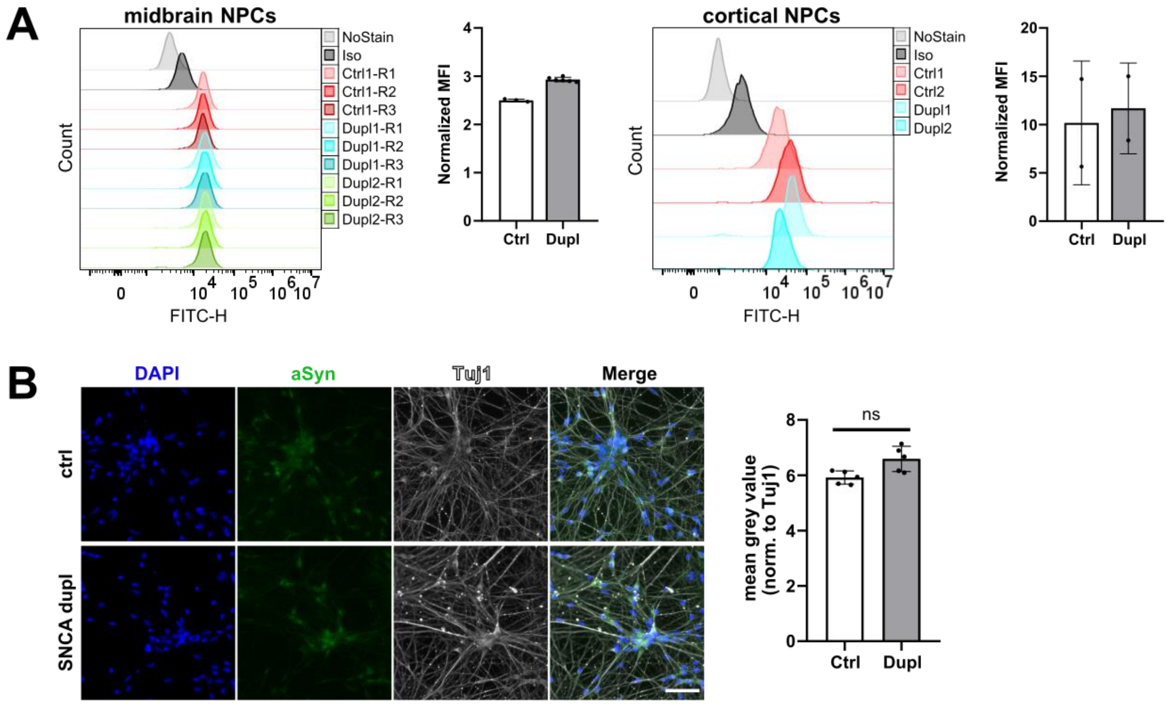

Supplementary Figure 4. The LB509 antibody does not detect physiological differences in aSyn expression in neural precursors and neurons. (A) Intracellular aSyn staining with the LB509 antibody in midbrain and cortical neural precursor cells (NPC) revealed only a marginal detection of an aSyn protein increase in NPC from PD patients with SNCA locus duplication (Dupl1 and 2 lines) compared to control NPC (Ctrl1 and 2 lines). Histograms ("midbrain NPCs" and "cortical NPCs") and quantified mean fluorescence intensities (MFI, right to each histogram) are shown. Normalized MFI values for each staining were calculated as a difference between MFI of antibody staining and of isotype control staining (Iso). **(B)** The LB509 antibody does not detect a significant increase of aSyn expression in hiPSC-derived midbrain dopaminergic neurons from SNCA locus duplication Parkinson's disease patients (SNCA dupl) compared to control (ctrl) using immunocytochemistry. Left: Representative images of ctrl (Ctrl1 line) and SNCA dupl hiPSC-derived (Dupl1 line) midbrain dopaminergic neurons stained for aSyn (green) with LB509 antibody, tubulin β3 (Tuj1; white), and DAPI (blue). Right: Quantification of mean grey values of the LB509 aSyn staining of ctrl and SNCA dupl midbrain dopaminergic neurons. Error bars represent standard deviations. 5 independent measurements were performed. ns - not significant, one-way ANOVA.
